# Entropy-driven binding of gut bacterial β-glucuronidase inhibitors ameliorates irinotecan-induced toxicity

**DOI:** 10.1038/s42003-021-01815-w

**Published:** 2021-03-04

**Authors:** Hsien-Ya Lin, Chia-Yu Chen, Ting-Chien Lin, Lun-Fu Yeh, Wei-Che Hsieh, Shijay Gao, Pierre-Alain Burnouf, Bing-Mae Chen, Tung-Ju Hsieh, Punsaldulam Dashnyam, Yen-Hsi Kuo, Zhijay Tu, Steve R. Roffler, Chun-Hung Lin

**Affiliations:** 1 Institute of Biological Chemistry, Academia Sinica, Taipei, Taiwan; 2 Department of Chemistry, National Taiwan University, Taipei, Taiwan; 3 Institute of Biomedical Sciences, Academia Sinica, Taipei, Taiwan; 4 Graduate Institute of Medicine, College of Medicine, Kaohsiung Medical University, Kaohsiung, Taiwan; 5 Institute of Biochemical Sciences, National Taiwan University, Taipei, Taiwan; 6 The Genomics Research Center, Academia Sinica, Taipei, Taiwan

**Keywords:** X-ray crystallography, Hydrolases, Small molecules, Structure-based drug design, Thermodynamics

## Abstract

Irinotecan inhibits cell proliferation and thus is used for the primary treatment of colorectal cancer. Metabolism of irinotecan involves incorporation of β-glucuronic acid to facilitate excretion. During transit of the glucuronidated product through the gastrointestinal tract, an induced upregulation of gut microbial β-glucuronidase (GUS) activity may cause severe diarrhea and thus force many patients to stop treatment. We herein report the development of uronic isofagomine (UIFG) derivatives that act as general, potent inhibitors of bacterial GUSs, especially those of *Escherichia coli* and *Clostridium perfringens*. The best inhibitor, C6-nonyl UIFG, is 23,300-fold more selective for *E. coli* GUS than for human GUS (*K*_*i*_ = 0.0045 and 105 μM, respectively). Structural evidence indicated that the loss of coordinated water molecules, with the consequent increase in entropy, contributes to the high affinity and selectivity for bacterial GUSs. The inhibitors also effectively reduced irinotecan-induced diarrhea in mice without damaging intestinal epithelial cells.

## Abstract (web summary)

Hsien-Ya Lin, Chia-Yu Chen, Ting-Chien Lin and colleagues perform structure-guided modifications of the compound uronic isofagomaine in order to engineer a highly specific and potent inhibitor of gut bacterial β-glucuronidases (GUSs). The authors present eight crystal structures and demonstrate in vivo efficacy of the optimised C6-alkyl derivative inhibitor in mice models. This study may enhance the development of inhibitors of microbial GUS for use in colorectal cancer therapy to minimize the undesired side effects of irinotecan treatment.

## Introduction

Colorectal cancer is the third most common cancer worldwide^1^. More than half of colorectal cancer patients are diagnosed at stage 2 or later and therefore must receive chemotherapy. Irinotecan is the first-line chemotherapeutic agent for treatment of metastatic colorectal cancer^2–4^, and SN-38 is its active metabolite that blocks DNA replication by inhibiting type I topoisomerase, leading to cell death^5,6^. SN-38 is converted to SN-38-glucuronide by UDP-glucuronosyltransferase in the liver to facilitate drug excretion. However, microflora-encoding β-glucuronidases (GUSs) are notorious for reversing the glucuronidation to release SN-38 in the intestinal lumen and thus represent the major cause of undesirable effects. Among patients taking irinotecan, 87% suffer from severe delayed diarrhea^7–9^, and ~10% become dehydrated, whereas ~3.5% are at high risk of death owing to neutropenia^10,11^.

Glucuronidation is one of the most common biological conjugation reactions^12–14^. The monosaccharide glucuronic acid can be attached to an oxygen, nitrogen, or sulfur atom of substrates, catalyzed by UDP-glucuronosyltransferases, rendering the resulting metabolites easily excreted via urine or bile^15^. However, intestinal bacterial GUSs cleave the attached glucuronic acid for use as a carbon source, allowing potentially toxic compounds to re-enter the enterohepatic circulation^16,17^. Furthermore, several food-derived toxic compounds are released back into the bloodstream by these GUSs, such as amygdalin (found in apricots, peaches, bitter almonds, plums)^18^, 2-amino-3-methylimidazo[4,5-f]quinoline (genotoxic/carcinogenic compound formed in meat and fish during cooking)^19^, and bis(2-ethylhexyl)phthalate and its derivatives (plasticizers)^12^. These toxic compounds can promote tumor formation^20,21^, although they can be metabolized by UDP-glucuronosyltransferase to produce harmless glucuronide conjugates.

Several approaches have been proposed to suppress the irinotecan-induced intestinal toxicity, including enhanced the delivery of SN-38 to tumor^22^, adjusted the releasing rate of SN-38^23^, and reduced the immune response^24,25^. Another promising approach was to develop specific inhibitor of intestinal bacterial GUSs^9,26,27^. Redinbo and coworkers were the first to report a selective inhibitor (ASN03273363) for gut bacterial GUSs (*K*_*i*_ = 0.16 µM for *Escherichia coli* GUS, *Ec*GUS) that does not inhibit human GUS (*Hs*GUS). ASN03273363 can alleviate the undesirable effects caused by SN-38^9^. The *Hs*GUS and *Ec*GUS structures differ in loop 3, which is one of eight loops near the active site. Loop 3 of *Ec*GUS has 23 residues, 17 more than that of *Hs*GUS and interacts with ASN03273363 through hydrophobic contacts, explaining the potency and selectivity of ASN03273363. Despite its potency, ASN03273363 has one therapeutic drawback, namely that more than half of the microbial homologs have a loop 3 of <15 residues (Supplementary Fig. 1) and thus cannot interact with this inhibitor^28^. Moreover, the loop 3 sequence differs substantially among the various bacterial GUSs.

Uronic isofagomine (UIFG, **1**) potently inhibits both mammalian and bacterial GUSs, owing to ionic interactions between the protonated ring nitrogen and the two catalytic glutamates^29^. Notably, we found that the incorporation of an alkyl substituent to UIFG could affect the binding affinity with different GUSs. To understand the underlying structural basis, we prepared C6-propyl, -hexyl, and -nonyl UIFGs (**2**–**4**, respectively) and resolved the eight X-ray crystal structures of **1**–**4** bound to *Ec*GUS or *Bifidobacterium dentium* GUS (*Bd*GUS). C6-alkylation expelled the water molecules in the GUS active site, suggesting that the corresponding entropy increase contributes to the enhanced selectivity (*K*_*i*_ = 4.5 nM for *Ec*GUS vs. 105 μM for *Hs*GUS, a difference of 23,300-fold). To demonstrate in vivo efficacy, the inhibitors were tested for cytotoxicity and their ability to inhibit bacterial GUSs in the mouse intestine. The results indicated that C6-alkyl UIFG derivatives hold great promise for therapeutic intervention.

## Results

### Synthesis of inhibitors 2–4

**Fig. 1:**
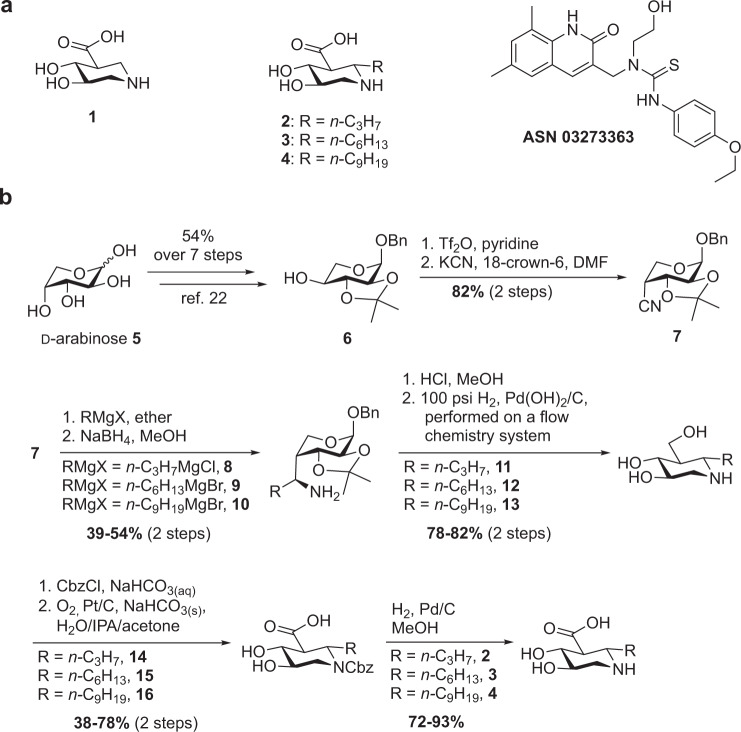
Molecular structures of inhibitors and synthetic scheme for UIFG derivatives. **a** Molecular structures of UIFG (**1**) and its C6-alkylated derivatives **2**–**4** and ASN03273363. **b** Synthesis of **2**–**4** and substituted isofagomines **11**–**13**.

GUS contains a deep enzyme active site (the two catalytic glutamates lie ~12 Å below the protein surface). To determine whether additional interactions with drugs could be achieved, we introduced three alkyl substituents (propyl, hexyl, nonyl) to C6 of UIFG (**2**–**4**, see Fig. 1a for the structures). The synthesis began with inexpensive d-arabinose (Fig. 1b). Alcohol **6** was prepared at large scale (>10 g) in seven steps with 54% overall yield following a procedure modified from Stick et al.^30^. The triflate, obtained by treating compound **6** with triflic anhydride in pyridine, was further converted into the cyanide **7** using KCN as the nucleophile in the presence of 18-crown-6 (82% yield in two steps). The nucleophilic addition of *n-*C_3_H_7_MgCl in diethyl ether to **7**, followed by NaBH_4_-mediated reduction, gave the corresponding amine **8** (39% yield in two steps) with exclusive stereoselectivity (*S*-configuration for the newly formed stereogenic center)^30,31^. The perfect stereoselectivity was realized owing to the coordination of the magnesium (of the Grignard reagent) with the ring oxygen of **7**^31^.

In a similar manner, amines **9** and **10** were obtained as single isomers at 54% and 49% yield, respectively. However, the next-step reductive amination failed to reach a satisfying yield when compounds **8**–**10** were subjected to hydrogenation by following the condition from our previous study (in the presence of conc. HCl over 20% Pd(OH)_2_ on charcoal at 50 psi H_2_)^29^. Thin-layer chromatography (TLC) analysis indicated that the reaction bottleneck was the formation of the cyclic imine. The reduction was achieved when the hydrogenation process was performed in a continuous flow chemistry system (Vapourtec^®^) by well-controlled circulation of amines **8**–**10** and 100 psi H_2_ through pre-coated 20% Pd(OH)_2_/C. The desired substituted isofagomines **11**–**13** were obtained in high yield (78–82%). The final products **2**–**4** were obtained with total yield of 27–73% in three steps by *N*-Cbz protection, selective oxidation of the primary alcohol, and removal of Cbz by hydrogenation. Notably, the selective oxidation of the methyl hydroxyl group was the key step, avoiding our previous use of tedious protection and deprotection steps^29^. The selective oxidation of the primary alcohol could be achieved in moderate yield (38–78%) by bubbling oxygen through a mixture of 10% Pt/C and *N*-Cbz-protected isofagomines **11**–**13** under basic conditions in H_2_O/isopropanol/acetone (Fig. 1b).

### Inhibition of GUSs by UIFG (1) and its derivatives (2–4)

Among the eight loops near the catalytic site of bacterial GUSs, loops 3–5 have highly variable sequences, which underlie substrate preferences of the GUSs^32,33^. Especially, loops 3 and 4 often differ in length^33^. To examine if the synthesized inhibitors displayed any specificity for different GUSs, we prepared the GUSs from four different bacterial strains belonging to major phyla of the gut microbiota, including *Ec*GUS, *Bd*GUS, *Clostridium perfringens* GUS (*Cp*GUS), and *Lactobacillus gasseri* GUS (*Lg*GUS). *E. coli* and *C. perfringens* are opportunistic bacteria, whereas *B. dentium* and *L. gasseri* are commensal bacteria. Notably, *Ec*GUS, *Cp*GUS, and *Lg*GUS contain a longer loop 3 (>15 residues), whereas *Hs*GUS and *Bd*GUS both have only six residues in their loop 3. In addition, loop 4 of *Bd*GUS is five residues longer than that of the other GUSs (Supplementary Fig. 2). Comparison of sequences from different bacterial GUSs helped us to understand if the potency of GUS inhibition correlated with loop length, as well as their preference for xenobiotic glucuronides. We previously reported that the structures and conformations of loops 3 and 5 are closely linked to substrate preference^32^.

Table 1 lists the inhibition constants for inhibitors **1**–**4** for the five GUSs. Inhibitor **1** appeared to be potent for both human and bacterial GUSs (e.g., *K*_*i*_ = 180 nM for *Hs*GUS, 16 nM for *Ec*GUS, 7.4 nM for *Bd*GUS, 6.5 nM for *Cp*GUS). Surprisingly, the C6-substituted UIFG contributed to differential selectivity between bacterial and *Hs*GUS: the longer the C6-alkyl chain, the more potent the inhibition of microbial GUSs. Particularly, C6-nonyl UIFG (**4**) had 23,300-fold higher potency for *Ec*GUS than for *Hs*GUS (*K*_*i*_ = 4.5 nM for *Ec*GUS vs. 105 μM for *Hs*GUS). Though less potent for *Lg*GUS and *Bd*GUS, **4** still inhibited these two GUSs in the sub-micromolar range (*K*_*i*_ = 270 and 440 nM, respectively) and was selective for the two bacterial GUSs (390- and 240-fold more potent than for *Hs*GUS, respectively). Likewise, C6-hexyl UIFG (**3**) also was 1770-fold more potent for inhibiting *Ec*GUS than *Hs*GUS.

**Table 1:** Inhibition constants for UIFGs (**1**) and derivatives (**2**–**4**) for *Hs*GUS and four gut bacterial GUSs.

	*K*_*i*_ (μM)				
Enzyme	1	2	3	4	ASN03273363
*Hs*GUS	0.18 ± 0.002	36.1 ± 7.5	5.3 ± 0.9	105.2 ± 4.8	NI^a^
*Ec*GUS	0.016 ± 0.002	0.035 ± 0.009	0.003 ± 0.0006	0.0045 ± 0.0007	0.16^a^
*Cp*GUS	0.0065 ± 0.0012	0.62 ± 0.041	0.17 ± 0.066	0.026 ± 0.0073	0.97^a^
*Lg*GUS	0.11 ± 0.033	18.8 ± 3.92	0.86 ± 0.18	0.27 ± 0.074	NI
*Bd*GUS	0.0074 ± 0.002	4.9 ± 1.2	0.86 ± 0.2	0.44 ± 0.02	NI

*NI* no inhibition.

^a^Ref. ^20^.

Table 1 also illustrates a notable trend. In comparison with **1**, the introduction of an alkyl chain indeed decreased affinity for all GUSs, yet there was a trend of increasing affinity with longer chain length. This compensation effect was more important for the inhibition of *Ec*GUS and *Cp*GUS. To help explain these intriguing observations, we relied on the structures of inhibitor-bound *Ec*GUS and *Bd*GUS. Additionally, we also examined the inhibition of isofagomines **11**–**13** that are the analogs of **2**–**4**, respectively. The main difference is that **11**–**13** contain a hydroxymethyl group at C5, instead of a carboxylic acid. Despite a much lower level of inhibition for **11**–**13** (IC_50_ = 272.3, 24.9, and 3.6 µM for *Ec*GUS, respectively), these compounds displayed a consistent trend observed for inhibitors **2**–**4**, i.e., a longer alkyl chain could improve the inhibition of *Ec*GUS. This trend was reversed, however, for *Hs*GUS: the percentage of inhibition = 4.5%, 0.6%, and –0.5% for **11**–**13** at 1 mM (IC_50_ > 1 mM), respectively.

### The alkyl chain of 2–4 disrupts the water-mediated hydrogen-bond network at the inhibitor binding site

**Fig. 2:**
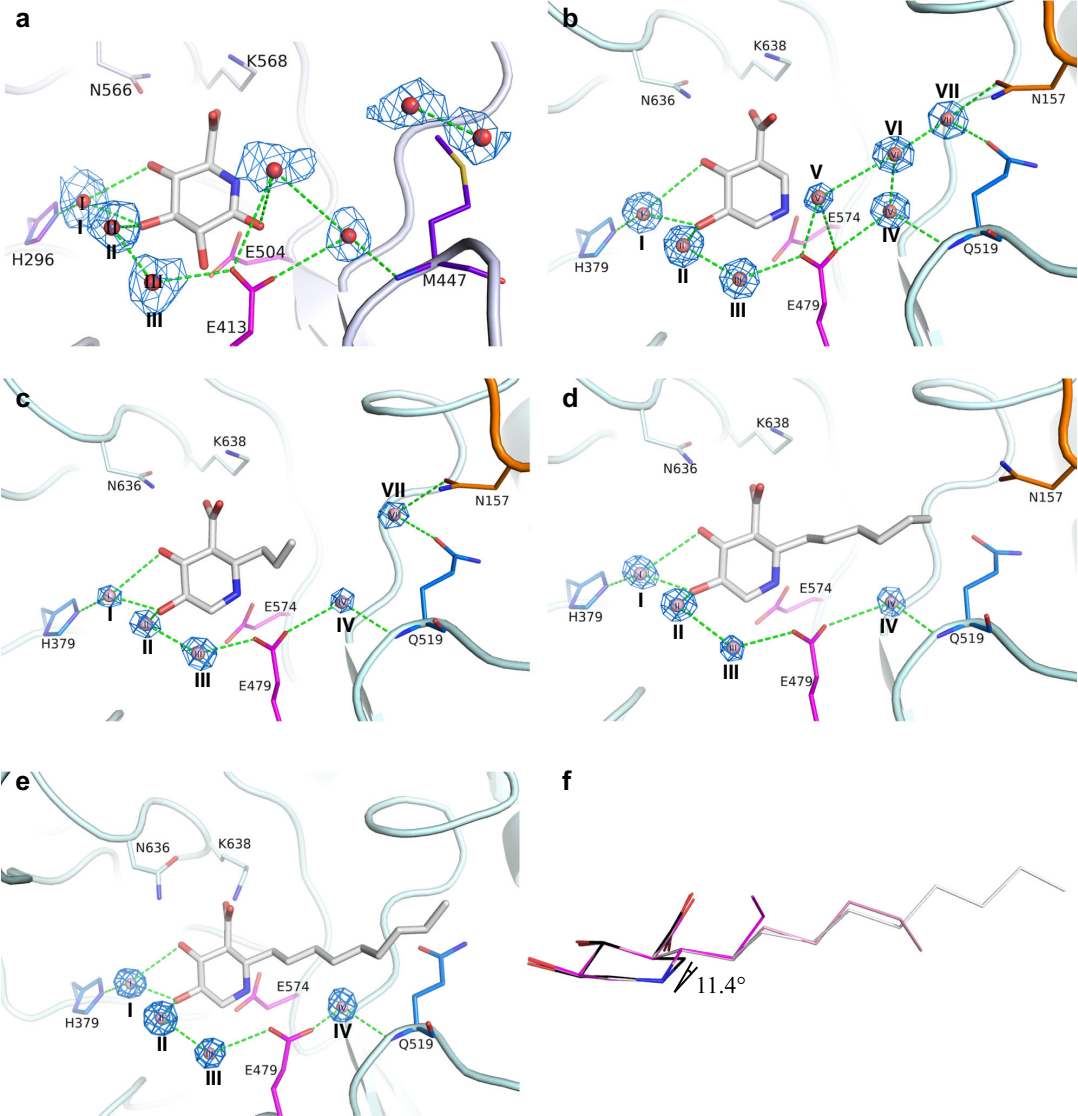
Crystal structures of inhibitors bound to *Bd*GUS. A water-mediated H-bond network within the substrate binding site of **a** *Ec*GUS in complex with glucaro-δ-lactam and **b**–**e** *Bd*GUS in complex with inhibitors **1**–**4**. The interactions were similar between the sugar moiety and *Bd*GUS. The 2*F*_o_*–F*_c_ density maps of water molecules are colored blue. The alkyl substituents of **2**–**4** appear to expel some of the water molecules. **f** Superimposition of inhibitors **1**–**4** in the crystal structures. C6 of **2**–**4** deviates 11.4° from that of **1**.

To resolve the details of the binding interactions, we determined the complete structures for inhibitors **1**–**4** bound to *Ec*GUS and *Bd*GUS, (Fig. 2b–e and Supplementary Fig. 3). These structures displayed binding interactions similar to those with the UIFG moiety, which is equivalent to glucuronic acid (the reaction product). For *Bd*GUS, the two catalytic residues Glu479 and Glu574 (equivalent to Glu413 and Glu504 in *Ec*GUS that correspond to the acid/base and nucleophile, respectively) formed ionic interactions with the endocyclic amine of UIFG^29^. Asn636 and Lys638, characterized as a unique conserved N–K motif in loop 8^33^, provided hydrogen bonds (H-bonds) and electrostatic interactions, respectively, with the C5-carboxylate of inhibitors **1**–**4** (Fig. 2b–e). The other conserved residues were categorized into two groups depending on whether they formed direct or indirect H-bonds with the hydroxyl groups of the bound inhibitor. The former group (including Arg632, Asp179, and Tyr543) interacted directly with the C5-carboxylate and C4-hydroxyl groups of the inhibitors, whereas the latter (His379, His413, and Asn478) formed water-mediated H-bonds with the C3-OH and Glu574. Additionally, Trp619 provided a hydrophobic contact (Supplementary Fig. 4) with one face of the iminocyclitol ring. Because the resolutions of the four structures for *Bd*GUS (1.7–2.4 Å) were higher than those for *Ec*GUS (2.5–3.2 Å), water molecules could be visualized in the catalytic site. Seven highly coordinated water molecules (I–VII) clustered in the active site of *Bd*GUS/**1** and formed an H-bond network with the inhibitor. Water molecules I–III were conserved in all of the *Bd*GUS structures and located near the UIFG-binding moiety to mediate interactions between the inhibitor and certain residues. In contrast, molecules VI–VII were located near the aglycone-binding moiety.

As compared with the inhibitor **1**-bound form, two water molecules (V and VI) were not present in the structure of **2**-bound *Bd*GUS, likely owing to the presence of the propyl substituent of **2**. Interestingly, waters V–VII were absent in the inhibitors **3**- and **4**-bound forms. Although water molecules were ambiguous in the active site of *Ec*GUS (apo form) owing to the limited resolution, in the glucaro-δ-lactam-bound structure of *Ec*GUS (PDB code: 3K4D)^9^, we indeed observed five water molecules that also formed an H-bond network similar to what was observed in the inhibitor-bound structures of *Bd*GUS (Fig. 2a, b).

**Fig. 3:**
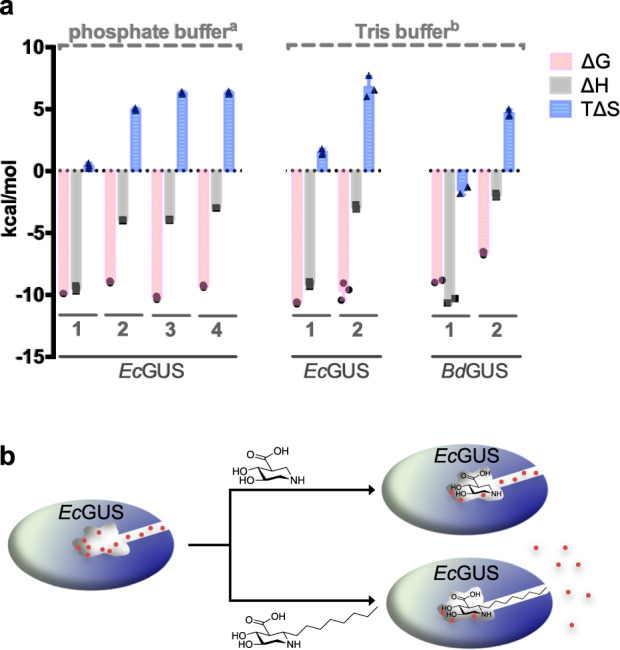
Entropy-driven binding of inhibitors 1–4 with *Ec*GUS and *Bd*GUS. **a** Graphical presentation to show the thermodynamic parameters of the binding interactions of *Ec*GUS and *Bd*GUS in complex with inhibitors **1**–**4** at 298 K. The detailed analysis is shown in Supplementary Table 1. ^a^ 250 mM NaCl, 20 mM KH_2_PO_4_, 100 mM Na_2_HPO_4_, pH 8.0. ^b^ 250 mM NaCl, 20 mM Tris, pH 8.0. **b** Cartoon presentation to demonstrate that several water molecules (represented by red circles) are orderly located at the active site of gut bacterial GUSs, as shown in *Ec*GUS (the left model), and can be displaced by binding with UIFGs. The best inhibitor, C6-nonyl UIFG, displays tight binding with *Ec*GUS (*K*_*i*_ = 4.5 nM) and 23,300-fold higher selectivity for *Ec*GUS than for human GUS (the bottom right). GUSs are mainly different in the aglycone-binding site, which can be leveraged to develop selective inhibitors. If UIFGs have no substitution (the top right), selective inhibition no longer exists.

Isothermal titration calorimetry was used to measure the thermodynamics of the binding of **1** or **2** to *Ec*GUS and *Bd*GUS (Fig. 3a, Supplementary Table 1, and Supplementary Fig. 5). The potent inhibition of **1** was contributed mainly by enthalpy (Δ*H* = –10.47 and –9.12 kcal mol^−1^ for *Bd*GUS and *Ec*GUS, respectively). At pH 8.0, the endocyclic amine of inhibitor **2** (pKa = 9.4) was mostly protonated, which afforded a stronger electrostatic interaction with catalytic residues than did **1** (pKa = 8.0)^21^. The results for **2** were quite different. In addition to a small negative enthalpy change (about –2 to –4 kcal mol^−1^), binding was contributed mainly by entropy (+4.6 kcal mol^−1^ for *Bd*GUS in Tris buffer; +7.1 and +5.0 kcal mol^−1^ for *Ec*GUS in Tris and phosphate buffer, respectively). With the aforementioned structural information, the differences between the thermodynamic parameters for **1** and **2** support the idea that the lack of H-bonded water molecules for **2** diminished the coordinated H-bond network, which disfavored binding and thus the observed enthalpy penalty (ΔΔ*H*_**1**–**2**_ = +8.5 kcal mol^−1^ for *Bd*GUS in Tris buffer; +6.2 and +5.5 kcal mol^−1^ for *Ec*GUS in Tris and phosphate buffer, respectively). Furthermore, the expulsion of water molecules also correlated with the entropy increase (i.e., –*T*ΔΔ*S*_**1**–**2**_ = −6.2 kcal mol^−1^ for *Bd*GUS in Tris buffer; –5.6 and –4.6 kcal mol^−1^ for *Ec*GUS in Tris and phosphate buffer, respectively). Therefore, introduction of an alkyl substituent to C6 of UIFG produced entropy-driven binding. Further titration studies of the hexyl- and nonyl-substituted UIFGs (**3** and **4**) became impossible because the enthalpy change was close to zero in Tris buffer. It is known that protonation by Tris in solution makes the total process highly exothermic^34^; however, it may interfere with proton transfer in the binding reaction of inhibitor and GUS. Instead of Tris buffer, the use of phosphate buffer allowed measurement of the thermodynamic parameters. Similar to the previous trend, the entropy contribution of **3** and **4** increased with the length of alkyl chain (–*T*ΔΔ*S*_**2**–**3**_ = –1.32 kcal mol^−1^ and –*T*ΔΔ*S*_**2**–**4**_ = −1.34 kcal mol^−1^ for *Ec*GUS), while the enthalpy contribution decreased (ΔΔ*H*_**2**–**3**_ = +0.04 kcal mol^−1^ and ΔΔ*H*_**2**–**4**_ = +0.99 kcal mol^−1^ for *Ec*GUS). These results explain why the incorporation of an alkyl chain to C6 of UIFG reduced the binding affinity, but chain elongation increased the entropy contribution, thereby improving binding (Fig. 3b). Therefore, the water-mediated H-bond network likely plays an important role in both substrate/inhibitor binding to bacterial GUSs and their catalytic activity.

Water molecules were also reported in published structures of GUSs, such as *Bd*GUS (apo form), *Hs*GUS (apo form, PDB code: 3HN3), and *Ec*GUS (bound with glucaro-δ-lactam, PDB code: 3K4D). Notably, several water molecules in the apo structures overlap with the hydroxyl and carboxyl groups of glucuronic acid, implying that the sugar-binding site either interacts with the complexed waters (apo form) or with the glucuronic acid moiety (binding site occupied by the substrate/inhibitor), as shown in Supplementary Fig. 6.

Furthermore, Phe448 in the **2**-bound *Ec*GUS structure was found to rotate ~100° toward the active site to form a hydrophobic interaction^35^ with the propyl group (Supplementary Figs. 3e and 7), as compared to the same residue in **1**-bound *Ec*GUS. This change shortened the distance between the propyl group and the center of the phenyl ring of Phe448 by 1.4 Å, thus providing a better hydrophobic contact. Interestingly, owing to the longer alkyl chains of **3** and **4**, Phe448 rotated outward in *Ec*GUS/**3** and *Ec*GUS/**4** and thus increased the hydrophobic contact area. This additional hydrophobic interaction might have compensated for the binding energy loss owing to the absence of the water-mediated H-bond network, leading to the observed lower *K*_*i*_ values of **2**–**4** for *Ec*GUS compared with other GUSs. Furthermore, *Ec*GUS contains a narrower aglycone-binding site than *Lg*GUS and *Bd*GUS (Supplementary Fig. 8). The alkyl substituent of the inhibitors therefore facilitated an increase in hydrophobic contacts with nonpolar residues of loops 3 and 5 in *Ec*GUS, e.g., such as Leu361, Phe365 in loop 3, and Val446, Met447, and Phe448 in loop 5.

### Compounds 2–4 inhibit *Ec*GUS without affecting the survival of *E. coli*

To examine if inhibitors **2**–**4** could inhibit GUS activity in vivo, *E. coli* cells (OD_600_ = 1.89) were treated with each of **2**–**4** for 30 min, after which GUS activity was measured. The IC_50_ was as follows: **2**, 1.2 μM; **3**, 18.6 nM; and **4**, 3.69 nM (Supplementary Fig. 9a). Consistent with the aforementioned *K*_*i*_ values, **4** was the most potent inhibitor. The fact that intracellular GUS activity could be inhibited indicated that **2**–**4** were, in fact, able to cross the cell membrane. Additionally, to evaluate cell viability, *E. coli* cultures were treated with ampicillin (positive control), DMSO, or inhibitors **2**–**4** (100 μM) for 6 h. An agar-based colony-forming assay revealed that **2**–**4** did not significantly affect *E. coli* viability (Supplementary Fig. 9b).

### Compound 3 inhibits the activity of intestinal microbial GUSs in BALB/c mice

We evaluated the potential cytotoxicity of **3** in cultures of non-cancerous human mammary epithelial cells (H184b5f5/M10) and human fibroblasts (GM637). The cells were chosen as representatives of the intestinal mucosa, the lumen of which is composed of epithelia and fibroblasts. Cells were incubated with **3** (0.001–100 μM) for 24 h. No cytotoxicity was evident, even at 100 μM of **3**. In contrast, ASN03273363 at 100 μM inhibited the proliferation of both cell types by >50% (Supplementary Fig. 10a).

**Fig. 4:**
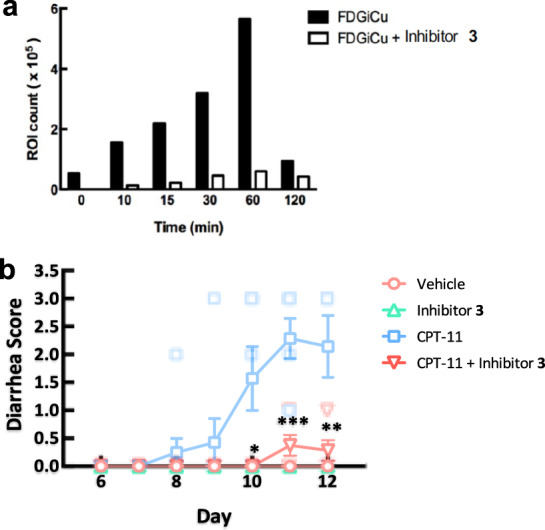
Treatment with inhibitor 3 demonstrates inhibition of GUS and protection against diarrhea caused by CPT-11 damage in mice. **a** Inhibition of GUS in vivo. Mice were given **3** via oral gavage. After 1 h, fluorescein-di-β-d-glucuronide (FDGiCu; 500 µg in 100 µL) was injected intravenously. The hydrolysis product (fluorescein) generated in the gut was quantified over a 2 h period by in vivo imaging (excitation 465 nm, emission 520 nm). The maximum fluorescein fluorescence was observed 60 min after injection of vehicle control. At 2 h post injection, most of the FDGiCu has been excreted and thus fluorescence is reduced. The region of interest (ROI) was analyzed with Living Image Software. **b** Effect of inhibitor **3** to protect against diarrhea caused by CPT-11. Diarrhea severity was scored as described in methods. Mice receiving CPT-11 (blue squares) experienced severe diarrhea from days 7–11, whereas mice receiving inhibitor **3** with CPT-11 (red triangles) displayed significantly reduced diarrhea (Welch’s unpaired *t*-test, day 9, *p* = 0.3632; day 10, *p = 0.0284; day 11, ***p = 0.0005; day 12, **p = 0.0057).

We also investigated the potential toxicity of **3** to intestinal cells using female BALB/c mice (8–12 weeks old). The mice were given **3** (37.5 mg per kg body weight) or ASN03273363 (65 mg) via oral gavage for 5 days. The mice were then sacrificed and the intestines removed. Hematoxylin and eosin staining revealed that neither compound affected the health of the epithelial layer or the intestinal glandular structure (Supplementary Fig. 10b). In addition, we performed real-time in vivo gastrointestinal inhibition of bacterial GUSs using whole-body in vivo imaging (Fig. 4a and Supplementary Figs. 11, 12). The mice were gavaged in the same manner as described above. Following the final gavage, 500 µg of FDGiCu (fluorescein-di-β-d-glucuronide as a non-fluorescent probe) was injected intravenously. Upon digestion by intestinal bacterial GUSs, FDGiCu is hydrolyzed to produce fluorescein (as the hydrolyzed product) that can be detected as fluorescence in the intestinal region. Reduced fluorescence was observed in mice fed with inhibitor **3**, suggesting that **3** could effectively inhibit gut bacterial GUSs. Moreover, to examine whether inhibitor **3** can block SN-38-induced toxicity by irinotecan (CPT-11), a total of 32 mice were divided into four groups for this study, including vehicle control, inhibitor **3** only (5.8 µg given orally twice per day), CPT-11 only (30 mg per kg intravenously daily), and CPT-11 with inhibitor **3** at the same doses as above. Mice were treated for 10 days. The results indicate that neither the negative control nor inhibitor **3** caused diarrhea in mice (Fig. 4b). The mice treated with CPT-11 developed diarrhea on day 7, until they reached a peak on day 11. In contrast, diarrhea was significantly suppressed when inhibitor **3** was given to mice receiving CPT-11.

## Discussion

In general, gut bacterial GUSs mostly contain a hydrophobic aglycone-binding site. Although some bacterial GUSs indeed have substrate preference for hydrophilic aglycones, those GUSs are secreted extracellularly to degrade polysaccharide glucuronides^33^. Because secreted GUSs are not xenotoxic, they were not the focus of our study. On the other hand, *Hs*GUS is a lysosomal enzyme that degrades glucuronate-containing glycosaminoglycans; its aglycone-binding site is quite hydrophilic.

Taking the comparison between *Ec*GUS and *Hs*GUS as an example, the aglycone-binding site of *Ec*GUS is surrounded by five hydrophobic residues, namely Phe365, Leu361, Val446, Met447, and Phe448, which are located in loops 3 and 5, whereas the site of *Hs*GUS is surrounded by Ser485^loop 5^, Tyr505^loop 6^, His509^loop 6^, and Thr599^loop 8^ (Supplementary Fig. 13a, b). This difference explains why substitution with an alkyl group at C6 of UIFG remarkably enhanced its binding affinity and selectivity for *Ec*GUS.

Our observed entropy-driven binding of inhibitors **2**–**4** to bacterial GUSs is reminiscent of the iminoribitol-based hydroxypyrrolidines that inhibit human 5ʹ-methylthioadenosine phosphorylase^36^. Schramm and coworkers reported that the binding involves a favorable entropy of –17.6 kcal mol^−1^ with unfavorable enthalpy of +2.6 kcal mol^−1^. The binding to the phosphorylase results in substantial structural changes, including collapse of the enzyme active site and the expulsion of water from both the active site and subunit interfaces^36^.

Moreover, because the aglycone-binding sites of *Cp*GUS and *EcGUS* are very similar in terms of hydrophobicity and size, it is not surprising that **2**–**4** could potently and selectively inhibit *Cp*GUS (Supplementary Figs. 8a and 7b). However, *Lg*GUS and *Bd*GUS have more spacious aglycone-binding sites than *Ec*GUS and *Cp*GUS (Supplementary Fig. 8b, c), explaining why **2**–**4** did not have high affinity for *Lg*GUS and *Bd*GUS. To develop inhibitors with higher affinity for *Lg*GUS and *Bd*GUS, a bulkier group should be considered, e.g., phenyl group. This also supports the idea that a functionalized substituent, such as an NH_2_-containing moiety, at C6 is essential for successful addition of any group at C6, depending on the features of the aglycone-binding site. Consequently, it is possible to develop potent and selective inhibitors for a particular type of GUS as long as the aglycone-binding site can be predicted in accordance with the sequences of loops 3–5.

In conclusion, we identified a water-mediated H-bond network in the catalytic site of bacterial GUSs. C6-substituted UIFGs were found to be entropy-favored inhibitors that achieved potent and selective inhibition of *Ec*GUS and *Cp*GUS, especially the enzymes produced by opportunistic and pathogenic bacteria that are major causes of xenobiotic toxicity. Moreover, the inhibitors we developed not only effectively inhibited gut bacterial GUSs in mice but also were not cytotoxic to the gut bacteria or intestinal epithelia. Because substituted iminocyclitols (such as Miglitol) are widely used for therapeutic intervention, our findings pave the way for iminocyclitols to be utilized in microbiota research and for clinical intervention.

## Methods

### Preparation of GUSs

*Hs*GUS was prepared as described^37^. Recombinant *Ec*GUS was cloned into pET-28a (Novagen), and *Cp*GUS, *Lg*GUS, and *Bd*GUS were cloned into pET-15b (Novagen). All proteins were overexpressed in *E. coli* BL21 (DE3) upon induction with 0.5 mM isopropyl β-d-1-thiogalactoside pyranoside for 24 h at 16 °C. Recombinant proteins were purified to homogeneity by Ni^2+^-affinity and size-exclusion chromatography. Purified proteins were then stored in gel-filtration buffer (20 mM succinic acid pH 6.0, 150 mM NaCl for *Bd*GUS; 20 mM Tris-HCl pH 8.0, 250 mM NaCl for *Ec*GUS, *Lg*GUS, and *Cp*GUS) and concentrated to 20 mg mL^–1^ as determined by the Bradford method.

### Measurement of IC_50_ and *K*_*i*_ values

GUS activities were determined by the hydrolysis rate of 4-methylumbelliferyl-β-glucuronide. Emission at 445 nm was monitored using an excitation wavelength of 365 nm to measure the release of fluorescent 4-methylumbelliferone at 37 °C. Enzyme kinetics were measured in 50 mM HEPES pH 7.5 for *Bd*GUS and *Ec*GUS and 50 mM sodium acetate pH 4.5 for *Hs*GUS, *Lg*GUS, and *Cp*GUS. To measure *K*_*i*_ values, the activity assays were carried out in 100 μL of the aforementioned buffers containing 1 mM 4-methylumbelliferyl-β-glucuronide and varying concentrations of inhibitor. *Bd*GUS (6.9 nM), *Ec*GUS (2.5 nM), *Lg*GUS (4.0 nM), *Cp*GUS (3.8 nM), and H*s*GUS (7.5 nM) were used for the inhibition assays. The *K*_*i*_ values for inhibitors **1**–**4** and *Hs*GUS, *Lg*GUS, *Cp*GUS, and *Bd*GUS were verified by Lineweaver–Burk plot^29^, and apparent *K*_*M*_ values were calculated. Plotting the apparent *K*_*M*_ values as a function of the inhibitor concentrations generated the secondary plot. *K*_*i*_ was determined by calculating the negative value of the resulting *x* intercept. As previously reported, the progression curves for inhibitors **1**–**4** and *Ec*GUS revealed time-dependent inhibition^29,38^, and *K*_*i*_ values were determined by the following simple reversible slow-binding equation:

1 $${\mathrm{E}} + {\mathrm{I}}\ \mathop{\leftrightarrows}\limits_{{k_2}}^{{k_1}}\ {\mathrm{EI}}\ \mathop{\leftrightarrows}\limits_{{k_4}}^{{k_3}}\ {\mathrm{EI}}^ \ast$$

2 $$P = V_{\mathrm{s}} \times t + \left( {V_0 - V_{\mathrm{s}}} \right)\frac{{\left( {1 - {\mathrm{e}}^{ - kt}} \right)}}{{k_{{\mathrm{obs}}}}}$$

where *V*_0_ and *V*_s_ are the initial and steady-state rates, respectively, *k*_obs_ is the apparent rate constant for the steady state, and *P* is the amount of product that accumulates during a period of time *t*.

3 $$k_{\mathrm{obs}} = k_3\left[ I \right] + k_4$$

The *k*_obs_ values were plotted as a function of inhibitor concentration. The linear fit of the data provided the kinetic rate constants *k*_3_ and *k*_4_, and then the apparent *K*_*i*_ was calculated from the ratio of *k*_4_*/k*_3_.

### Crystallization and data collection

Crystals of *Ec*GUS and *Bd*GUS were grown at room temperature (298 K) using the hanging-drop vapor diffusion method. The crystallization conditions were as follows: 20 mg mL^–1^ *Ec*GUS in 2 μL of 0.2 M MgCl_2_, 0.1 M Tris-HCl, pH 8.5, 21% (w/v) PEG 4K; 5 mg mL^–1^ *Bd*GUS in 2 μL of 0.1 M sodium cacodylate, pH 6.5, 8% (w/v) PEG 20K. Crystals of inhibitor/*Bd*GUS and inhibitor/*Ec*GUS were obtained by soaking. First, compounds **1**–**4** were dissolved in 100% DMSO to a concentration of 20 mM. For ligand soaking, crystals were transferred into the reservoir solution containing a compound concentration of 5 mM, incubating for 5 min prior to cryocooling. After 5 min, the crystals were transferred into reservoir solution containing 20% glycerol as a cryoprotector. The crystals were then flash-frozen in liquid nitrogen and stored for data collection. Table 2 summarizes the data statistics.

**Table 2:** Summary of crystal structure determination and refinement.

	*Bd*GUS				
	Apo form	Inhibitor 1	Inhibitor 2	Inhibitor 3	Inhibitor 4
PDB code	6LD6	6LDB	6LDD	6LDO	6LDC
*Data collection*					
Space group	P2_1_	P2_1_	P2_1_	P2_1_	P2_1_
Cell dimensions					
*a*, *b*, *c* (Å)	92.583, 104.902, 160.62	92.69, 104.64, 160.504	92.606, 105.928, 161.89	92.645, 104.77, 161.044	93.142, 104.814, 160.819
*α*, *β*, *γ* (°)	90, 91.16, 90	90, 91.29, 90	90, 91.33, 90	90, 91.22, 90	90, 91.08, 90
Resolution (Å)	29.6–2.204 (2.283–2.204)	24.85–1.651 (1.71–1.651)	29.59–2.449 (2.536–2.449)	26.6–1.881 (1.948–1.881)	27.84–2.175 (2.253–2.175)
*R*_sym_ or *R*_merge_	0.086 (0.385)	0.054 (0.418)	0.088 (0.471)	0.089 (0.513)	0.055 (0.189)
*Ι* / *σΙ*	18.9 (4.3)	22.0 (3.8)	14.8 (2.9)	20.1 (2.5)	23.8 (5.7)
Completeness (%)	98.9 (90.3)	93.8 (90.4)	93.14 (46.62)	92.33 (60.9)	97.08 (77.86)
Redundancy	4.3 (4.0)	3.4 (3.4)	3.7 (3.8)	3.4 (4.2)	4.1 (2.9)
*Refinement*					
Resolution (Å)	29.6–2.204 (2.283–2.204)	24.85–1.651 (1.71–1.651)	29.59–2.449 (2.536–2.449)	26.6–1.881 (1.948–1.881)	27.84–2.175 (2.253–2.175)
No. reflections	154377	343510	155860	241740	160237
*R*_work_ / *R*_free_	0.1519/0.1991	0.1555/0.1825	0.1443/0.2056	0.1419/0.1747	0.1426/0.1912
No. atoms					
Protein	19636	19660	19636	19636	19552
Ligand/ion	0	44	56	68	80
Water	1935	2689	1949	1608	2030
*B*-factors	22.80	21.31	23.60	27.70	21.20
Protein	22.10	19.91	23.50	26.40	20.20
Ligand/ion		20.00	25.70	35.10	24.50
Water	30.00	31.57	25.90	38.60	29.90
R.m.s. deviations					
Bond lengths (Å)	0.008	0.015	0.008	0.007	0.007
Bond angles (°)	1.11	1.36	1.14	1.10	1.06
Ramachandran favored (%)	97	97	96	97	97
Ramachandran outliers (%)	0	0.041	0.12	0	0.042

	*Ec*GUS			
	Inhibitor 1	Inhibitor 2	Inhibitor 3	Inhibitor 4
PDB code	6LEG	6LEJ	6LEL	6LEM
*Data collection*				
Space group	C2	C2	C2	C2
Cell dimensions				
*a*, *b*, *c* (Å)	207.527, 75.926, 168.281	167.686, 76.551, 125.426	165.781, 76.72, 124.888	68.361, 76.404, 126.4
*α*, *β*, *γ* (°)	90, 96.79, 90	90, 124.85, 90	90, 124.636, 90	90, 124.966, 90
Resolution (Å)	29.79–2.603 (2.696–2.603)	29.75–2.617 (2.711–2.617)	29.47–2.498 (2.588–2.498)	28.25–3.188 (3.302–3.188)
*R*_sym_ or *R*_merge_	0.179 (0.897)	0.092 (0.642)	0.111 (0.763)	0.172 (0.953)
*Ι* / *σΙ*	13.3 (1.5)	17.8 (2.0)	15.7 (1.6)	9.7 (1.1)
Completeness (%)	99.2 (94.7)	97.9 (89.1)	97.9 (90.3)	98.4 (91.2)
Redundancy	3.4 (3.4)	5.3 (4.6)	3.6 (2.8)	6.0 (2.9)
*Refinement*				
Resolution (Å)	29.79–2.603 (2.696–2.603)	29.75–2.617 (2.711–2.617)	29.47–2.498 (2.588–2.498)	28.25–3.188 (3.302–3.188)
No. reflections	79253	61283	43895	21727
*R*_work_ / *R*_free_	0.2317/0.2877	0.2367/0.2795	0.2378/0.2868	0.2108/0.2695
No. atoms				
Protein	19223	9507	9521	9446
Ligand/ion	44	28	34	40
Water	231	65	125	0
*B*-factors	51.32	57.16	58.33	77.73
Protein	51.54	57.36	58.74	77.75
Ligand/ion	30.99	36.81	32.28	71.98
Water	36.23	36.94	33.90	
R.m.s. deviations				
Bond lengths (Å)	0.008	0.012	0.013	0.003
Bond angles (°)	1.04	1.53	1.64	0.66
Ramachandran favored (%)	93	92	93	90
Ramachandran outliers (%)	0.97	0.77	0.94	1.12

### Determination and refinement of the crystal structures

The crystal structures of all complexes were solved by molecular replacement with PHENIX AutoMR using the published *Ec*GUS apo structure as the starting search model (PDB entry 3K46). Modeling was performed with PHENIX AutoBuild. Structures underwent multiple rounds of manual rebuilding and refinement with Coot and PHENIX. The figures were generated in Pymol.

### Isothermal titration calorimetry

*Bd*GUS and *Ec*GUS were diluted to appropriate concentrations in dialysis buffer (20 mM Tris-HCl pH 8.0, 250 mM NaCl or 20 mM KH_2_PO_4_, 100 mM Na_2_HPO_4_, pH 8.0, 250 mM NaCl). All samples were passed through 0.22 μm filters (Millipore). Isothermal titration calorimetry was performed using the Auto-iTC200 (MicroCal, Northampton, MA) at 298 K. Inhibitors **1–4** were dissolved in a stock solution of DMSO. To avoid heating effects owing to different concentrations of DMSO in the syringe and protein solutions, 5% DMSO was added to the protein. Curve fitting of the experimental data was performed with Origin version 7.0 (MicroCal).

### Cells and animals

H184B5F5/M10 non-cancerous human mammary epithelial cells and GM637 non-cancerous human fibroblasts were obtained from the American Type Culture Collection (ATCC, Manassas, VA). The cells were cultured in RPMI supplemented with 10% bovine calf serum, 2.98 g L^–1^ HEPES, and 2 g L^–1^ NaHCO_3_ in a 5% CO_2_ humidified atmosphere at 37 °C. Female BALB/c mice (8–12 weeks old) were purchased from the National Laboratory Animal Center (Taipei, Taiwan). All animals were allowed free access to food and water, and experiments were done according to the standards of the United Kingdom Co-ordinating Committee on Cancer Research Guidelines for the Welfare of Animals in Experimental Neoplasia. All animal experiments were certified by Institute Animal Care and Use committee of Academia Sinica (ASIACUC), protocol ID: 12-07-384.

### In vitro cytotoxicity

To study the potential cytotoxicity of inhibitor **3** to normal cells, 10,000 cells per well were seeded in a 96-well plate and incubated at 37 °C overnight. The cells were then incubated with a graded concentration of **3** for 24 h, washed twice with sterile phosphate buffered saline, and incubated for 16 h in fresh medium containing 1 µCi [^3^H]thymidine per well. The cells were trypsinized and harvested on glass fiber filters, radioactivity was counted, and % inhibition was calculated as: [c.p.m. (sample) × 100/c.p.m. (control)].

### Colon histology

Mice were given an inhibitor (11.75 nmol in 100 µL) via oral gavage twice a day for 5 days. After the final gavage, the mice were sacrificed and the colon harvested and fixed in neutral buffered formalin. Colon samples were embedded in paraffin and stained with hematoxylin and eosin.

### In vivo imaging of GUS activity

Female BALB/c mice (8–12 weeks old) were given various amounts of an inhibitor via oral gavage 1 h before imaging. Following the final gavage, 500 µg of FDGiCu (Invitrogen) was injected intravenously. The intensity of hydrolyzed fluorescein was assessed by in vivo imaging system (Caliper Life Sciences) and quantified as GUS activity.

### In vivo diarrhea test

Irinotecan (CPT-11) was purchased from Sigma as a hydrochloride salt. For animal studies, CPT-11 was dissolved in double-distilled water as a stock solution (2.5 mg mL^−1^). The inhibitor **3** was dissolved in 100% DMSO (5.78 mg mL^−1^), then diluted with double-distilled water to 57.8 µg mL^−1^. Vehicle control mice received an equivalent volume of 1% DMSO in double-distilled water as the experimental groups. Thirty-two healthy female BALB/c mice (6–8 weeks old) were divided into four groups of eight mice each: (I) vehicle control group, animals receiving 250 µL of double-distilled water intraperitioneally (i.p.) and 100 µL of 1% DMSO solution by oral gavage twice per day; (II) inhibitor **3** group, 250 µL of double-distilled water i.p. and inhibitor **3** (100 µL) by oral gavage twice per day (10 h separation) starting on day-1; (III) CPT-11 group in which CPT-11 (30 mg kg^−1^) was injected i.p. once in the morning with oral gavage of vehicle control twice for 10 days, and (IV) CPT-11 + inhibitor **3** group in which CPT-11 (30 mg kg^−1^) was injected i.p. once in the morning for 10 days and inhibitor **3** (100 µL) was orally gavaged twice per day (10 h separation). Total injected volume was considered as equal for each mouse and all mice were carefully monitored. The degree of diarrhea was monitored daily and described by using a scoring system defined as: 0, normal stool; 1, slight perianal staining of the coat; 2, moderate perianal staining of the coat; 3, watery mucosal-infused stool with severe perianal staining of the coat.

### Statistics and reproducibility

No data were excluded from the analyses. Unless otherwise noted, all graphs depict mean ± SEM. Statistical significance was determined with Welch’s unpaired *t*-test. Biostatistical analyses were done with GraphPad software (GraphPad Prism 7, La Jolla, CA, USA).

### Reporting summary

Further information on research design is available in the Nature Research Reporting Summary linked to this article.

## Supplementary information

Supplementary Information

Description of Additional Supplementary Files

Supplementary Data 1

Supplementary Data 2

Reporting Summary

## Data Availability

The data that support the findings of this study are available from the corresponding author on reasonable request. All the data supporting the findings of this study are available in the Supplementary Information. The source data underlying Table 1 and Fig. 4b are provide as Supplementary Data 1 and 2. The coordinates of the crystal structures have been deposited to PDB and the entry numbers are 6LD6 (apo *Bd*GUS), 6LDB (*Bd*GUS/**1**), 6LDD (*Bd*GUS/**2**), 6LDO (*Bd*GUS/**3**), 6LDC (*Bd*GUS/**4**), 6LEG (*Ec*GUS/**1**), 6LEJ (*Ec*GUS/**2**), 6LEL (*Ec*GUS/**3**), and 6LEM (*Ec*GUS/**4**).
