# Supplementary material for: Entropy-driven binding of gut bacterial β-glucuronidase inhibitors ameliorates irinotecan-induced toxicity
Source: Commun Biol. 2021 Mar 4;4:280. doi: 10.1038/s42003-021-01815-w (PMC7933434; doi:10.1038/s42003-021-01815-w)
Supplement: Supplementary file 1 — Supplementary Information [file 42003_2021_1815_MOESM1_ESM.pdf]

Entropy-driven binding of gut bacterial β-glucuronidase inhibitors ameliorates irinotecan-induced toxicity

Hsien-Ya Lin[1,‡], Chia-Yu Chen[1,‡], Ting-Chien Lin[1,2,‡], Lun-Fu Yeh[1], Wei-Che Hsieh[1], Shijay Gao[1], Pierre-Alain Burnouf[3], Bing-Mae Chen[3], Tung-Ju Hsieh[1], Punsaldulam Dashnyam[1], Yen-Hsi Kuo[1], Zhijay Tu[1], Steve R. Roffler[3,4] * and Chun-Hung Lin[1,2,5,6,*]

[1] *Institute of Biological Chemistry, Academia Sinica, Taipei, 11529, Taiwan*

[2] *Department of Chemistry, National Taiwan University, Taipei, 10617, Taiwan*

[3] *Institute of Biomedical Sciences, Academia Sinica, Taipei, 11529, Taiwan*

[4] *Graduate Institute of Medicine, College of Medicine, Kaohsiung Medical University, Kaohsiung 80708, Taiwan*

[5] *Institute of Biochemical Sciences, National Taiwan University, Taipei, 10617, Taiwan*

[6] *The Genomics Research Center, Academia Sinica, Taipei, 11529, Taiwan*

* *Corresponding author*

‡ *These authors contributed equally to this work.*

E-mail: chunhung@gate.sinica.edu.tw; sroff@ibms.sinica.edu.tw

I. Supplementary Results

Supplementary Figures 1-13 S2
Supplementary table 1 S8

II. Supplementary Notes

General reagents and instruments S17
Chemical synthesis and characterization of compound **17**, **18**, **19**, **6**, **7** S18
General hydrogenation set-up inflow S21
Chemical synthesis and characterization of compound **8**–**13** S22
Chemical synthesis and characterization of compound **14–16**, **20–21** and **2–4** S24
Supplementary NMR spectra S27
Supplementary information references S34

a)

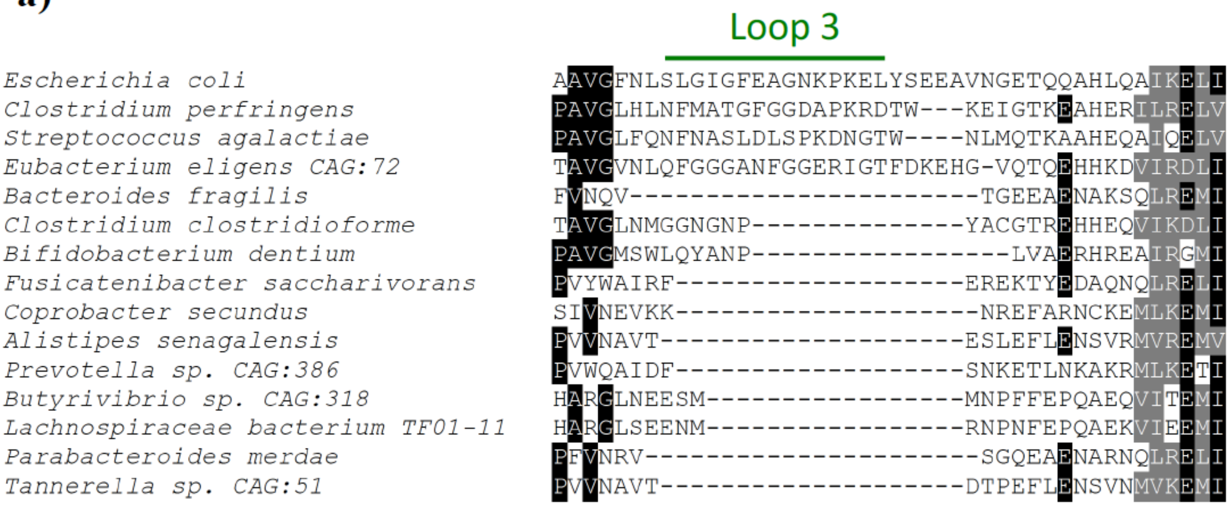

b)

Bacterial strain	Length of Loop 3	ASN03273363
Escherichia coli	15 aa	K_i = 0.16 μM[20]
Clostridium perfringens	15 aa	K_i = 0.97 μM[20]
Streptococcus agalactiae	15 aa	K_i = 1.4 μM[20]
Bacteroides fragilis	No	*NI*[20]
Clostridium clostridioforme	6 aa	*NI*
Bifidobacterium dentium	6 aa	*NI*

Supplementary Figure 1 ASN03273363 shown selective inhibition by targeting loops 3 in few GUSs. (a) Loop 3 of various GUSs. More than half of the microbial GUSs lack a long loop 3 (>15 residues), including GUSs from bacteria of the phyla Firmicutes, Bacteroidetes and Actinobacteria. The identical and similar residues in the multiple-alignment chart are represented by black and gray, respectively. (**b)** ASN03273363 inhibited only *Ec*GUS. aa: amino acid. *NI*: no inhibition.

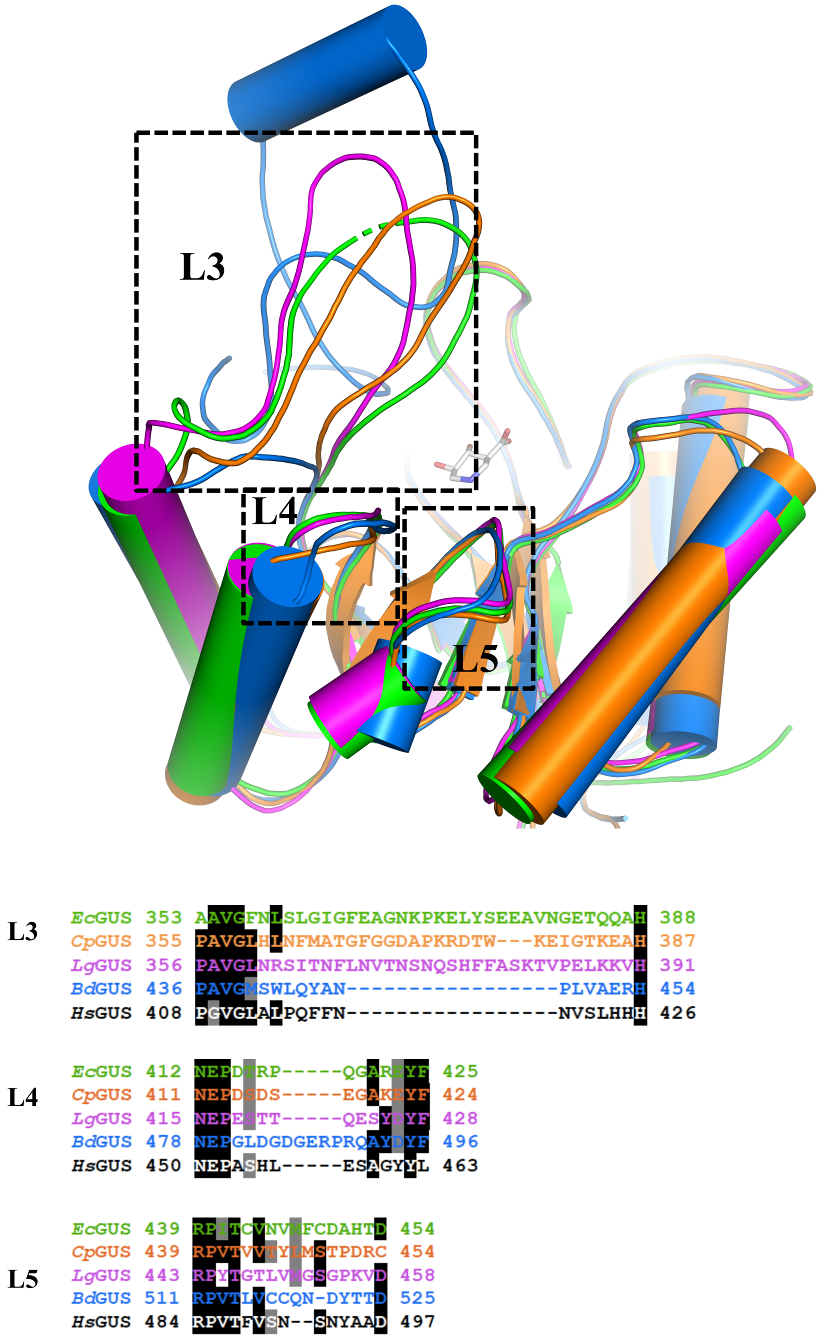

Supplementary Figure 2 Sequence alignment of loops 3–5 among for bacterial GUSs. Among the eight loops near the active site of GUSs, loops 3–5 are highly variable. *Bd*GUS (blue, PDB code: 6LD6), *Cp*GUS (orange, PDB code: 4JKM), *Lg*GUS (magenta, the modeling structure was built with Swiss model–based homology modeling[1] using *Ec*GUS as the template.) and *Ec*GUS (green, PDB code: 3K46).

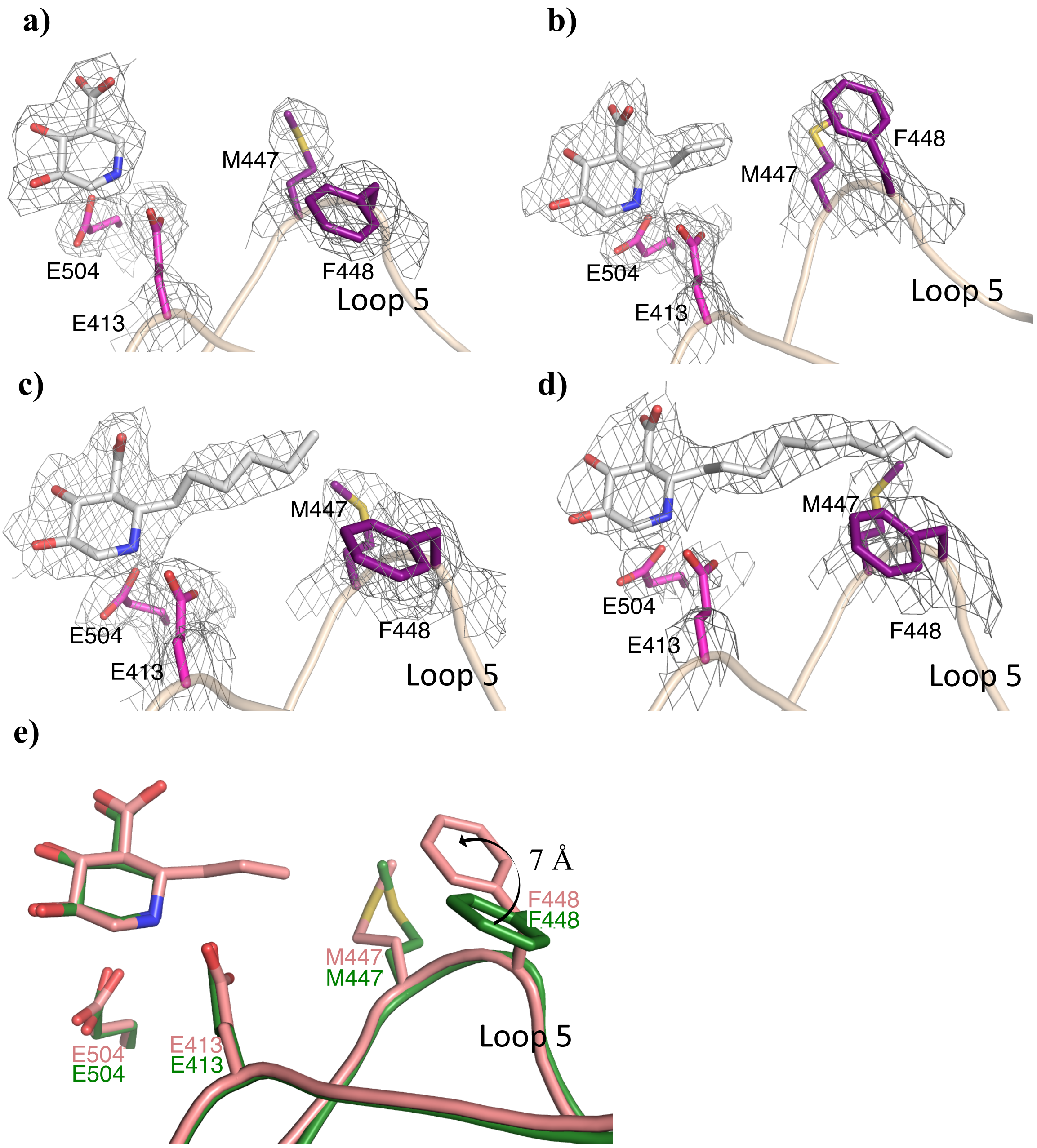

Supplementary Figure 3 Structures of *Ec*GUS bound to inhibitors 1–4. (a–d) Structures of *Ec*GUS bound to each of inhibitors **1–4**. The $2F_o$–F_c density maps are shown as a gray lattice. **(e)** Superimposition of *Ec*GUS/**1** (green) and *Ec*GUS/**2** (light pink) indicated that the binding of inhibitor **2** caused the inward rotation of the side chain of Phe448, thus forming a hydrophobic interaction with the propyl group of **2**.

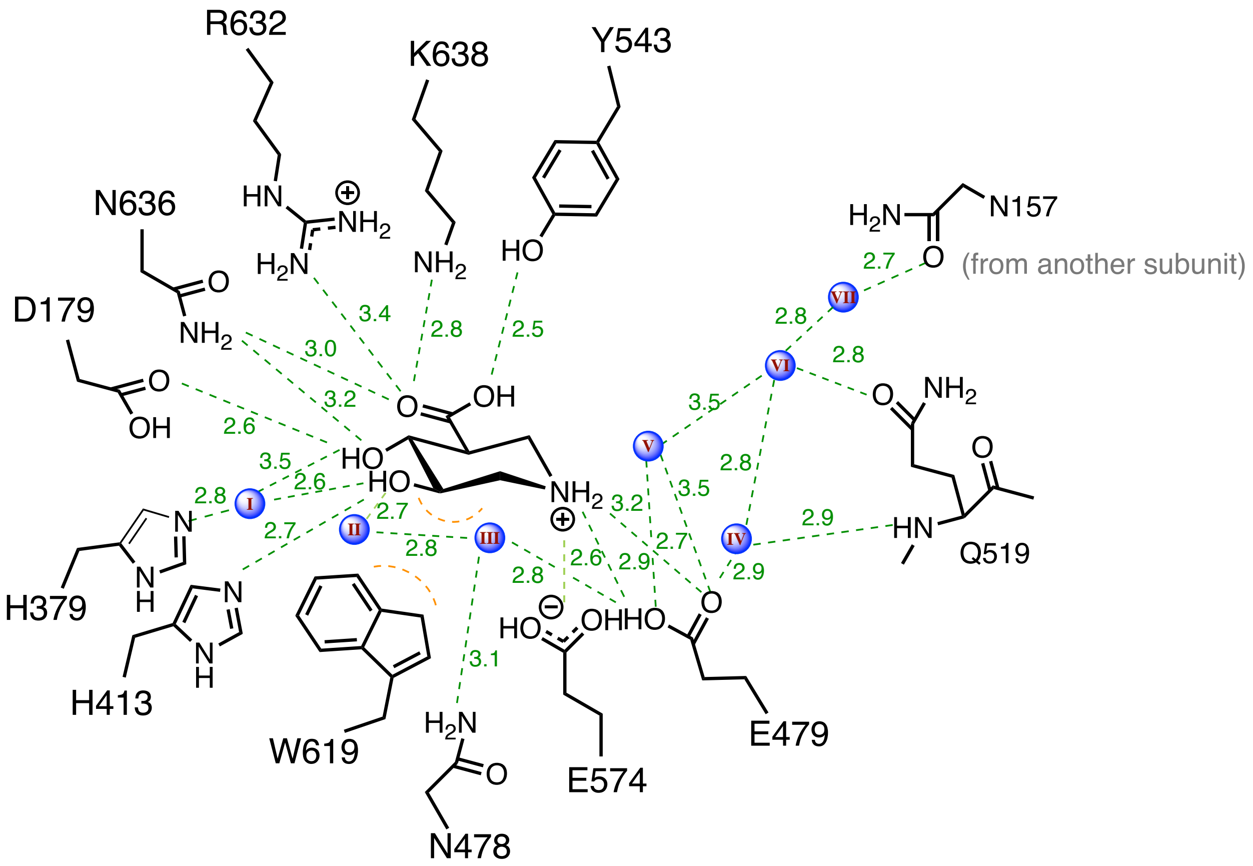

Supplementary Figure 4 Diagram of the *Bd*GUS active site with inhibitor 1 bound. Both direct and water-mediated H-bonds are labeled and shown as green dashed lines with bond lengths indicated. The hydrophobic contact formed between Trp619 (W619) and inhibitor **1** is illustrated by the dashed orange arc. The seven conserved water molecules I to VII are shown in blue.

Supplementary Table 1 Thermodynamic parameters of the binding interactions of *Ec*GUS and *Bd*GUS with inhibitor **1**–**4** at 298 K

	ΔG (kcal/mol)	ΔH (kcal/mol)	—TΔS (kcal/mol)	K_d (μM)
*Bd*GUS (Tris buffer[a])				
**1**	–8.91 ± 0.13	–10.47 ± 0.25	1.56 ± 0.37	0.30 ± 0.06
**2**	–6.60 ± 0.15	–1.97 ± 0.15	–4.64 ± 0.30	14.51 ± 3.55
*Ec*GUS (Tris buffer[a])				
**1**	–10.63 ± 0.075	–9.12 ± 0.14	–1.51 ± 0.20	0.02 ± 0.002
**2**	–10.01 ± 0.58	–2.90 ± 0.23	–7.11 ± 0.82	0.06 ± 0.0049
*Ec*GUS (phosphate buffer[b])				
**1**	–9.89 ± 0.01	–9.48 ± 0.29	–0.41 ± 0.30	0.06 ± 0.001
**2**	–8.96 ± 0.09	–3.99 ± 0.06	–4.97 ± 0.15	0.28 ± 0.05
**3**	–10.24 ± 0.18	–3.95 ± 0.06	–6.29 ± 0.13	0.03 ± 0.01
**4**	–9.30 ± 0.13	–3.00 ± 0.02	–6.31 ± 0.15	0.16 ± 0.04

[a] 250 mM NaCl, 20 mM Tris, pH 8.0. [b] 250 mM NaCl, 20 mM KH_2PO_4, 100 mM Na_2HPO_4, pH 8.0.

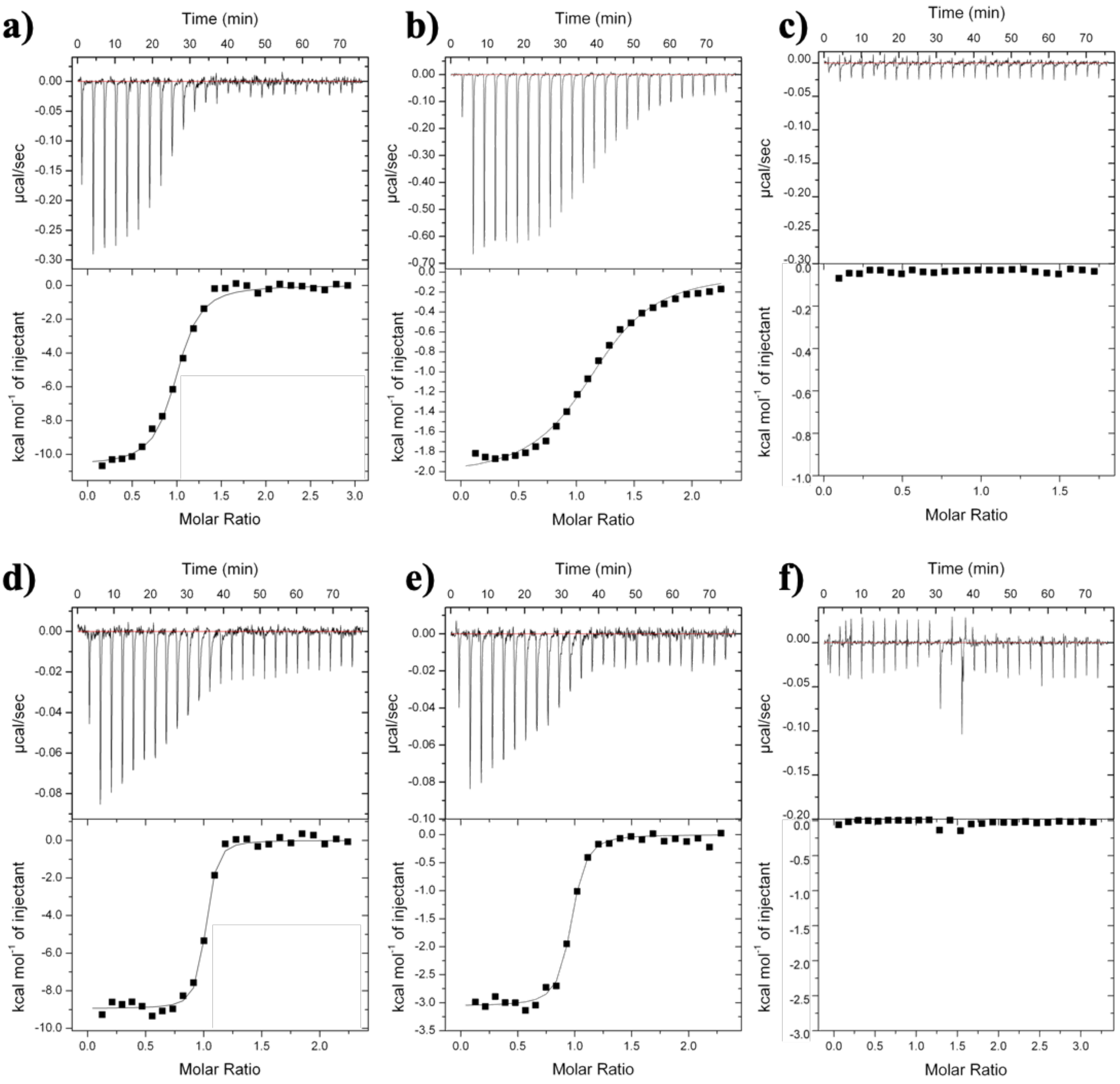

Supplementary Figure 5 Isothermal titration calorimetry plots for UIFGs 1–3 binding to *Bd*GUS (a–c) and *Ec*GUS (d–f). Experiments were conducted at 298 K in 20 mM Tris-HCl buffer (pH 8.0). Each top panel shows the injection profile, and each bottom panel shows the calorimetric binding isotherm that was created by plotting the integrated enthalpy peaks against the molar ratio of each UIFG. The exothermic reaction of **2** bound to GUS is much less than that of **1** bound to GUS. Essentially no enthalpy change was evident for **3** when **3** was titrated to the GUSs.

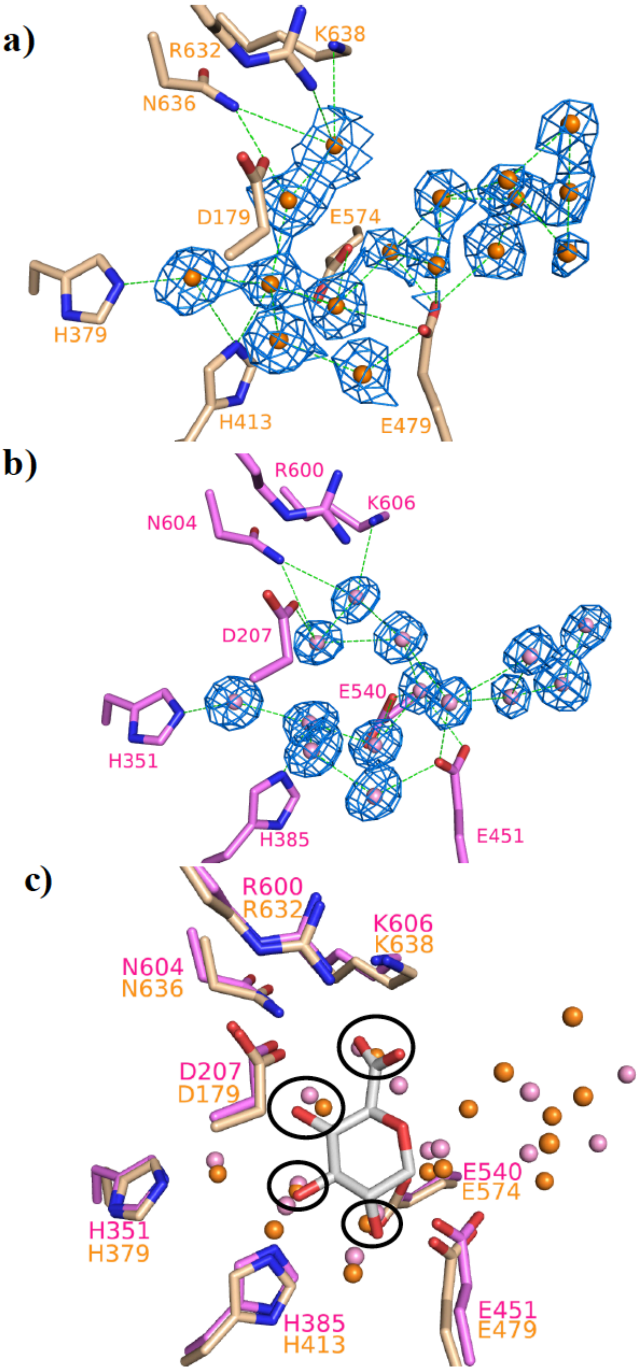

Supplementary Figure 6 Structures of the catalytic site with the water network. The water network of the catalytic site of *Bd*GUS (apo) **(a)** and *Hs*GUS (apo) **(b)**. **(c)** Superimposition of *Bd*GUS/glucuronic acid (PDB code: 5Z1B), *Bd*GUS (apo) and *Hs*GUS (apo). Black circles indicate water molecules that overlap with the carboxyl and hydroxyl groups of the glucuronic acid. Water molecules and conserved residues in the active site of *Bd*GUS and *Hs*GUS are colored orange and pink, respectively. Glucuronic acid is represented as stick and colored white.

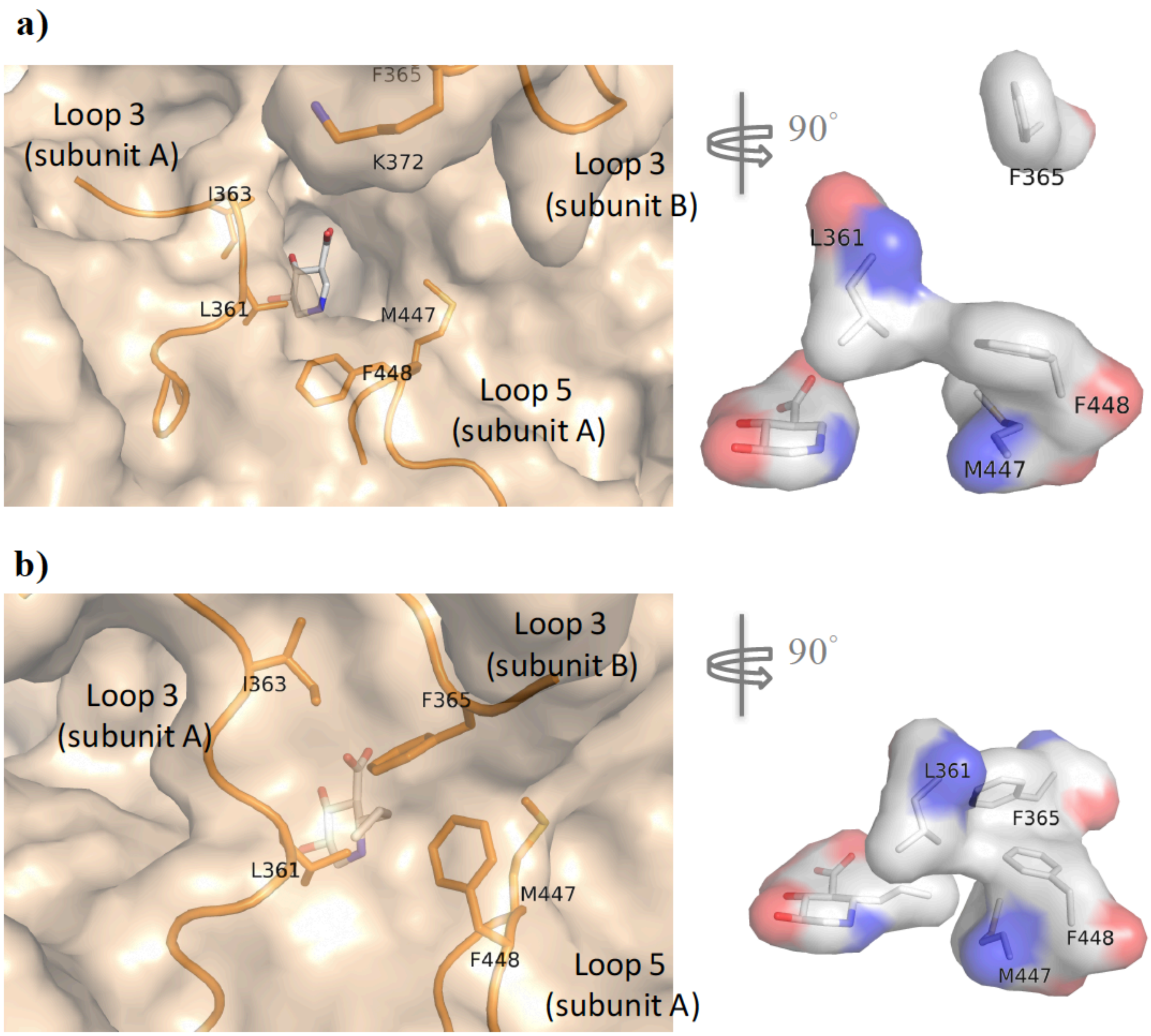

Supplementary Figure 7 The protein surface of *Ec*GUS bound to inhibitor 1 (a) and 2 (b). In comparison with the **1**-bound form, loops 3 and 5 of *Ec*GUS are closer to the catalytic site in the **2**-bound form, thus forming more hydrophobic interactions with the propyl side chain.

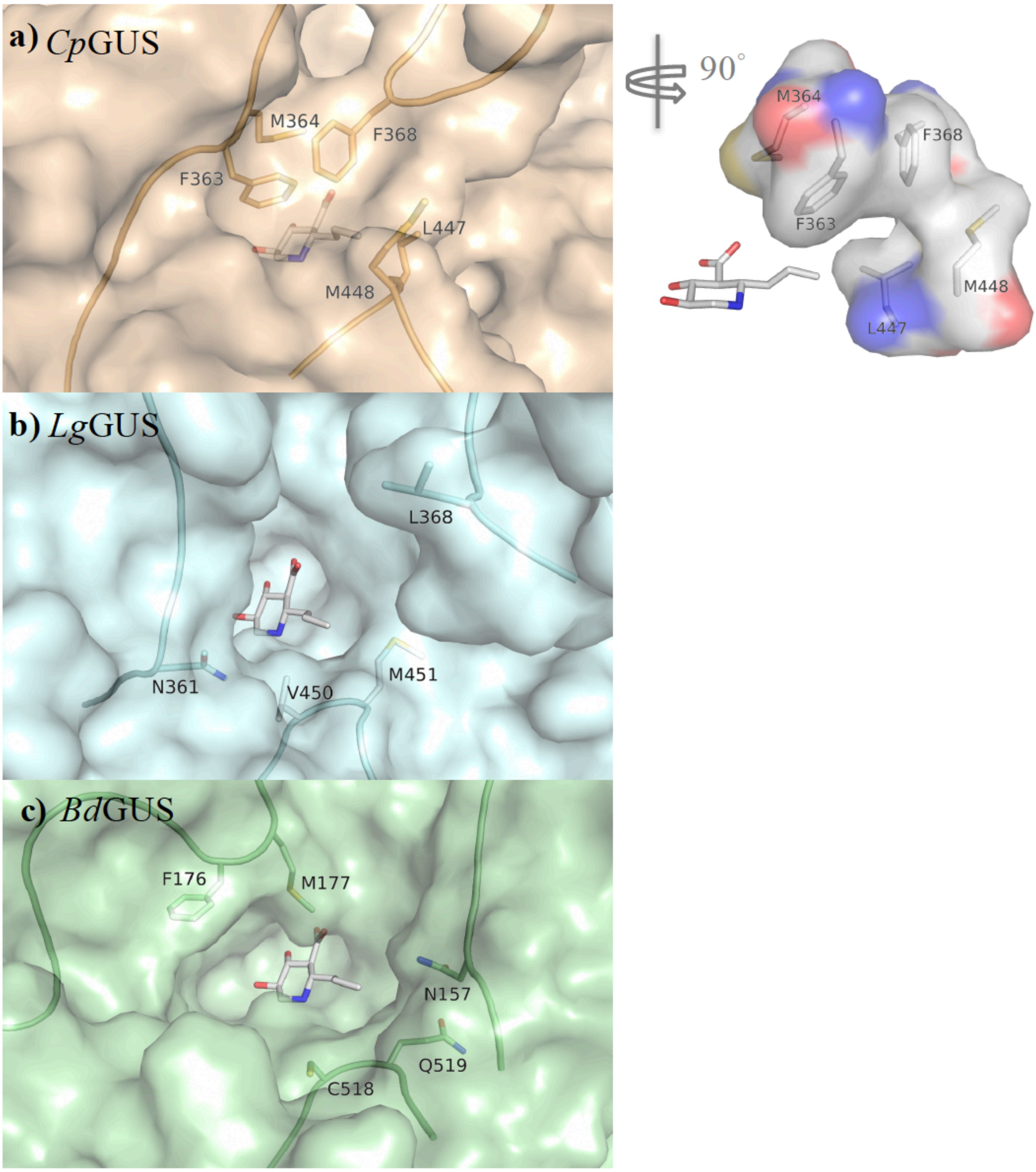

Supplementary Figure 8 The protein surface of (a) *Cp*GUS, (b) *Lg*GUS and (c) *Bd*GUS. The structure of *Lg*GUS was built with Swiss model–based homology modeling[1] using *Ec*GUS (PDB code: 3LPF) as the template. *Cp*GUS and *Lg*GUS were superimposed with the structure of *Ec*GUS/**2**. The catalytic site of *Cp*GUS is similar to that of *Ec*GUS, which is much narrower than that of *Lg*GUS and *Bd*GUS, thus providing more hydrophobic contacts with the alkyl substituent.

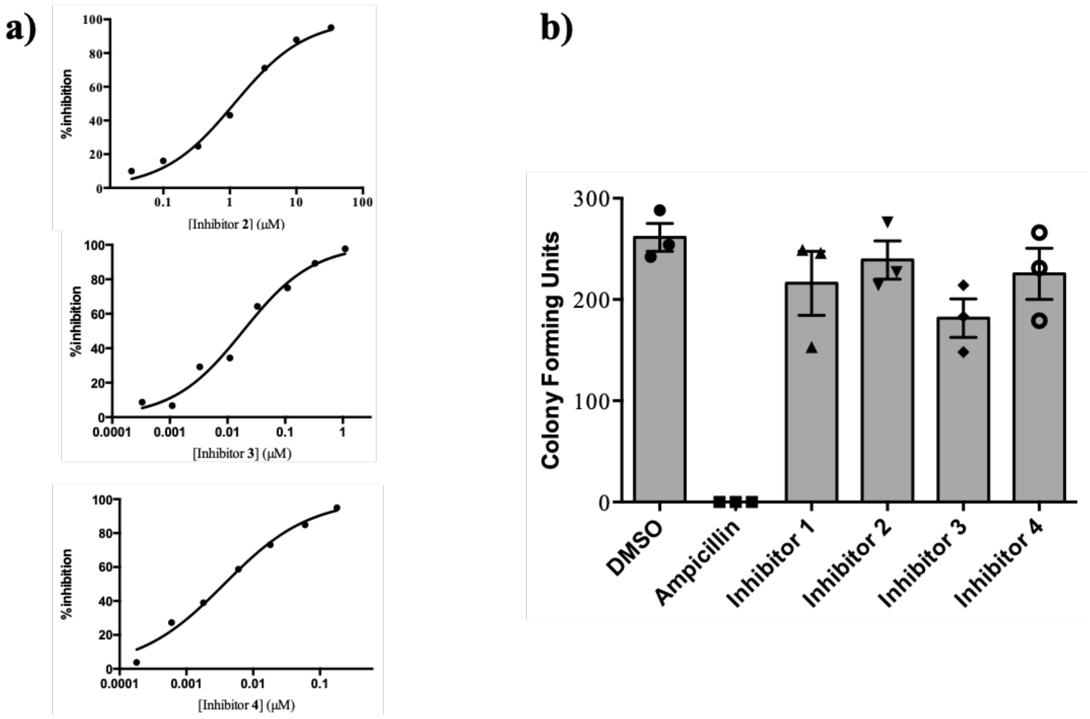

Supplementary Figure 9 Inhibitors 2–4 show inhibition against GUS activity in live *E. coli* cells. (a) GUS activity was measured in live *E. coli.* After *E. coli* were incubated with DMSO or an inhibitor (**2–4**), GUS activity was measured using 4-methylumbelliferyl-β-glucuronide as the substrate. The release of fluorescent 4-methylumbelliferone was detected at 445 nm (excitation, 345 nm). Compared with the negative control (DMSO), the IC_{50} was 1.2 μM (**2**), 18.6 nM (**3**), and 3.69 nM (**4**). **(b)** Colony formation assay. None of the inhibitors affected bacterial growth. As a negative control, *E. coli* cells were incubated with ampicillin (100 μg/mL). Results are expressed as the mean of triplicate samples ± SEM.

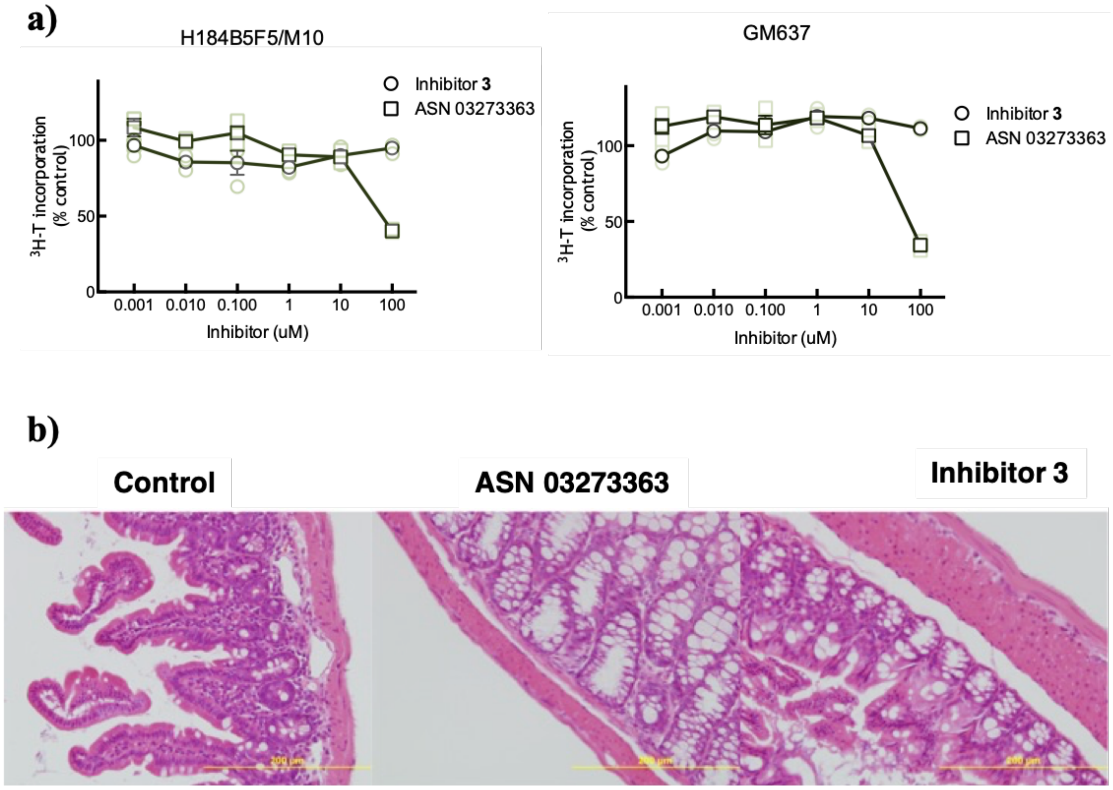

Supplementary Figure 10 The potential cytotoxicity of 3 in cultures of non-cancerous human mammary epithelial cells (H184b5f5/M10) and human fibroblasts (GM637). (a) Comparison of the cytotoxicity induced by inhibitors of bacterial GUSs. Noncancerous human mammary epithelial cells (H184B5F5/M10) and human fibroblasts (GM637) were seeded at 10,000 cells per well in a 96 well plate and incubated overnight. Cells were exposed to increasing concentrations of inhibitor **3** or ASN03273363 for a period of 24 h, and the relative viability of the cells was measured by [^{3}H]thymidine ([^{3}H]-T) incorporation. Results represent the mean of triplicate samples ± SEM. **(b)** Hematoxylin and eosin staining of mouse colon tissue. Mice were treated twice a day with 11.75 nmol of inhibitor for a period of 5 days. Tissue integrity was preserved in all treated mice (5 per group).

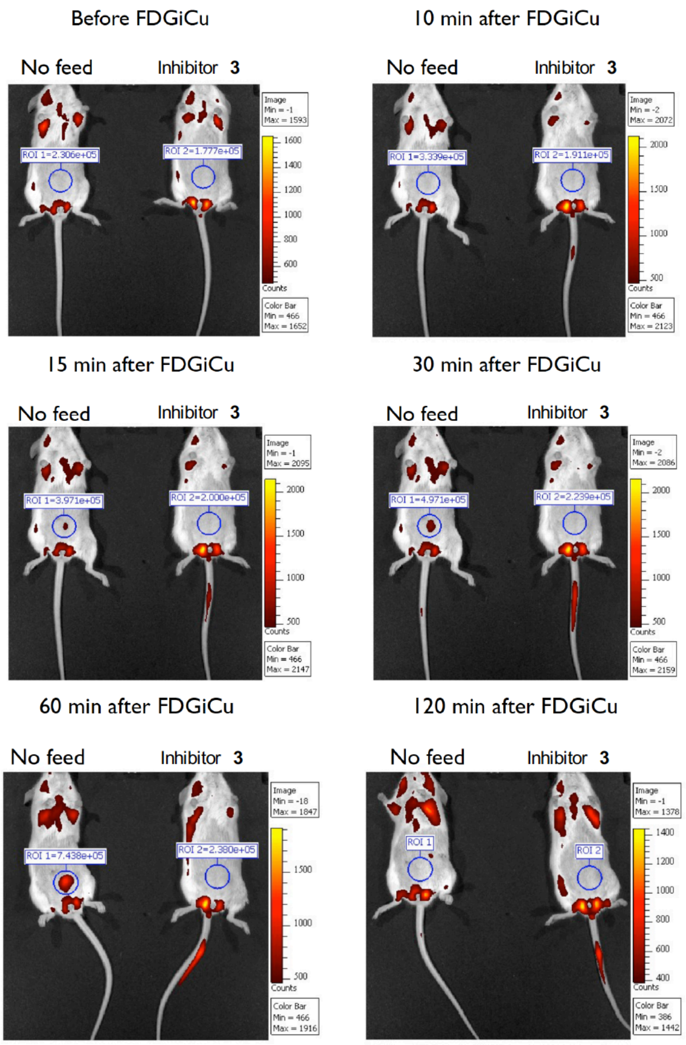

Supplementary Figure 11 *In vivo* imaging of intestinal GUS inhibition. Mice were fed with 12.3 µg inhibitor **3** via oral gavage. The hydrolysis product (fluorescein) generated in the gut was quantified over a 2 h period by *in vivo* imaging (excitation 465 nm, emission 520 nm).

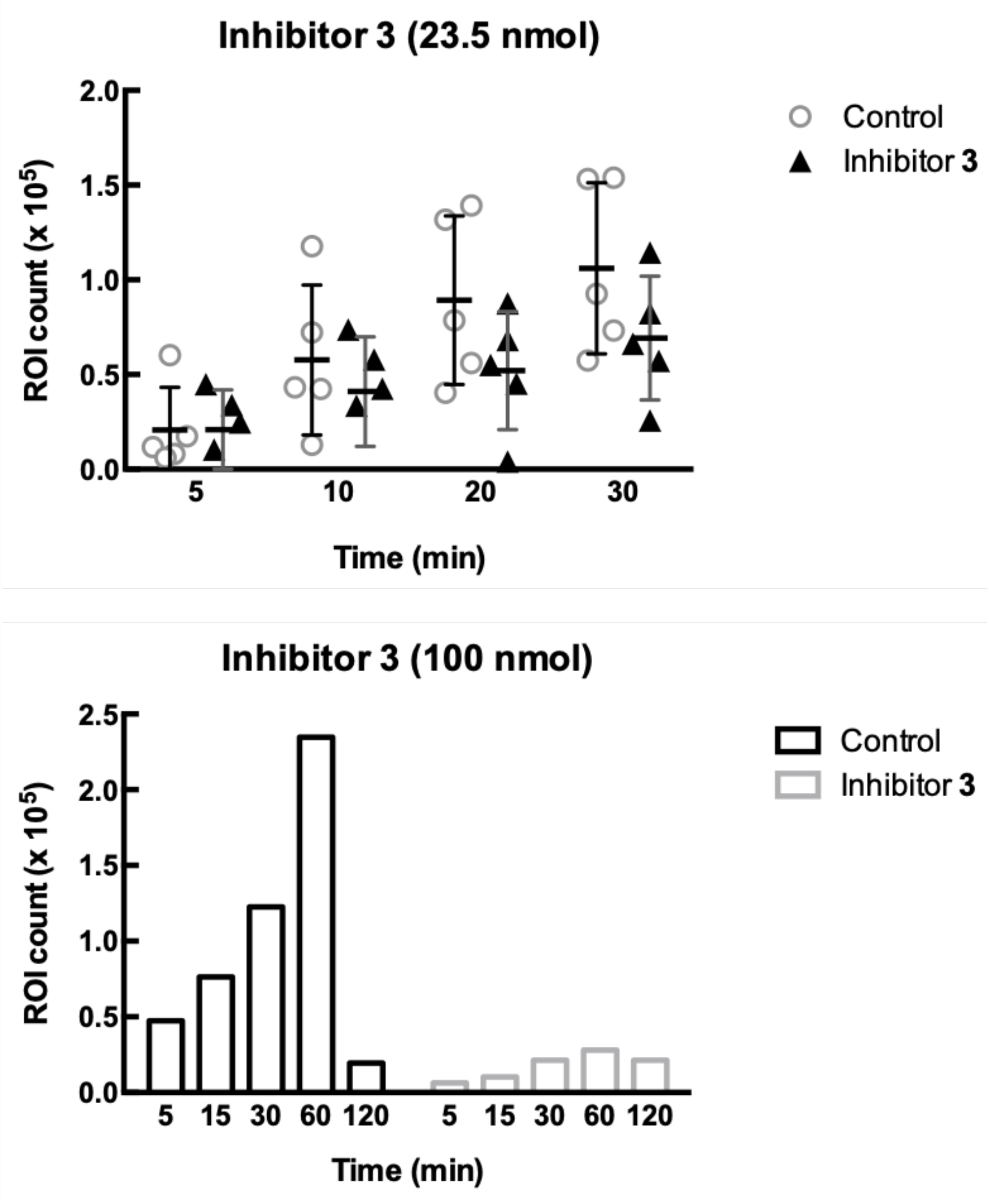

Supplementary Figure 12 Quantification of fluorescent signals from *in vivo* imaging system. Mice were given inhibitor **3** via oral gavage two times at the indicated dose (23.5 or 100 nmol). After the last gavage, fluoresceindiglucuronide (FDGiCu, 500 µg in 100 µL) was injected intravenously. The fluorescein generated in the gut was quantified by *in vivo* imaging at excitation 465 nm and emission 520 nm. Results are expressed as units of efficiency. N = 5 mice per group (upper panel), 1 mouse per group (lower panel). ROI, region of interest.

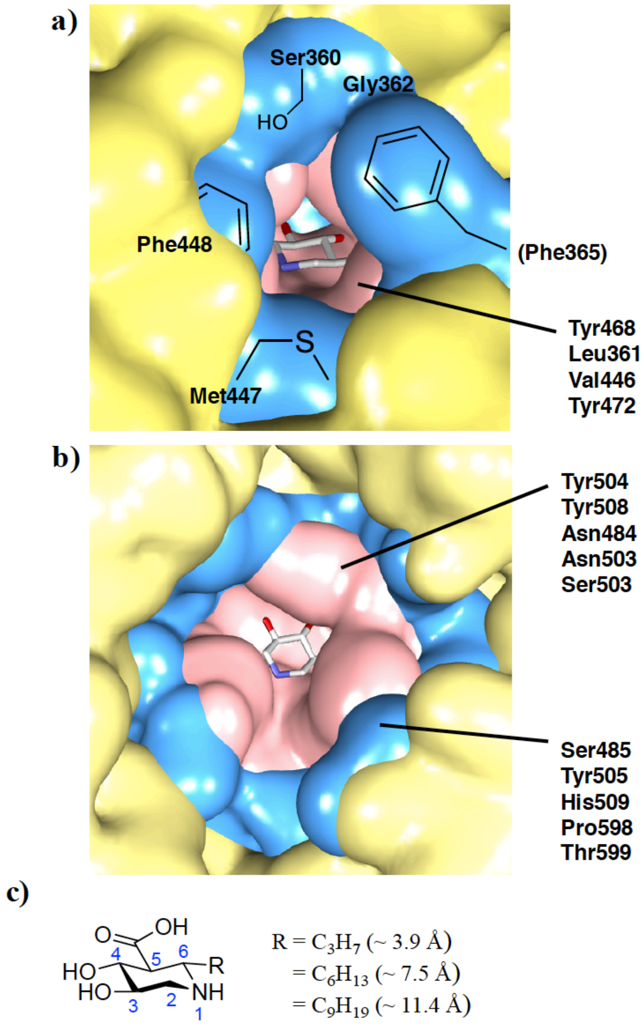

Supplementary Figure 13 Surface model of the substrate-binding site of each of (a) *Ec*GUS and (b) *Hs*GUS. The colors represent three ranges of distances from C6 of UIFG to the protein surface, including <8 Å (pink), 8–12 Å (blue), and >12 Å (yellow). Beyond a depth of 11.4 Å, a part of the substrate protrudes from the GUS surface. (**c**) The three alkyl chains attached to C6 of UIFG included propyl (C_3H_7), hexyl (C_6H_{13}), and nonyl (C_9H_{19}) groups.

General reagents and instruments

All chemicals were purchased form commercial supplies (Acros, Fluka, Sigma and Alfa Aesar) without further purification unless otherwise specified.

All reactions were conducted in oven-dried glassware, under nitrogen atmosphere. The solvents for extraction and chromatography were of ACS grade. Dichloromethane (DCM), tetrahydrofuran (THF), acetonitrile (ACN) were pre-dried by using molecular sieves and then percolated through an active Al_2O_3 column. Anhydrous N,N-dimethylformamide (DMF), pyridine, and methanol were purchased from chemical company directly and stored at electronic dry box.

The reaction products were purified by using column chromatography on silica gel (Geduran Silicagel 60, 0.040-0.063 mm, from Geduran®). Anhydrous solvents and moisture-sensitive materials were transferred by using an oven-dried syringe or cannula through a rubber septum. Organic solutions were concentrated under reduced pressure in a water bath (< 40 °C.). TLC was performed on pre-coated glass plates of TLC Silica gel 60G F254 (Merck KGaA®), and was detected by UV lamp (254 nm) and/or by staining reagents that contained ceric ammonium molybdate (for general use), *p*-anisaldehyde (for carbohydrates) or ninhydrin (for amino-group-containing samples).

1H and 13C NMR spectra were recorded on Bruker AV-400 (400 MHz) or AVII-500 (500 MHz) spectrometers by using tetramethylsilane (δ_H = 0.00 ppm), $CDCl_3$ (δ_H = 7.26 ppm), CD_3OD (δ_H = 3.31 ppm, central line of a quintet) or D_2O (δ_H = 4.80 ppm) as internal standards. ^{13}C NMR spectra were recorded on Bruker AV-400 (100 MHz) or AVII-500 (125 MHz) spectrometers by using $CDCl_3$ (δ_C = 77.2 ppm, central line of a triplet) or CD_3OD (δ_C = 49.2 ppm, central line of a septet) as internal standards. 2D NMR spectra (including ^1H-^1H COSY, ^1H-^{13}C HMQC, and NOESY) were acquired on Bruker AV-400 or AVII-500 spectrometers. High-resolution mass spectroscopy (HRMS) was performed on Bruker Bio-TOF III (ESI-TOF) spectrometers or Bruker Ultraflex (MALDI-TOF/TOF) and were reported as mass/charge (*m/z*) ratios with percentage relative abundance. Optical rotations were measured at the sodium D-line (589 nm) at 20 °C or 25 °C on a PerkinElmer® Model 341 Polarimeter. Specific rotations were reported as $[\alpha]^{20}_D$ or $[\alpha]^{25}_D$ after dividing the observed values by the sample concentration (*C*, in g mL^{-1}) and the path length (*l*, in dm).

Chemical synthesis and characterization of compound 17, 18, 19, 6, 7.[2-3]

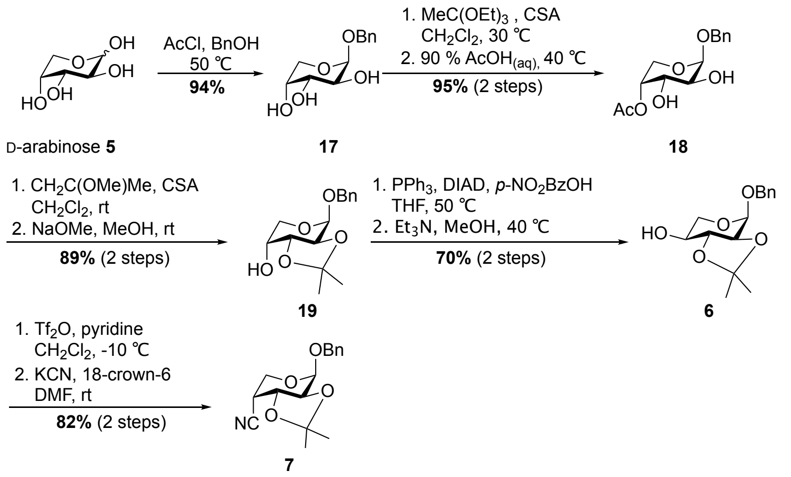

Scheme S1. Synthetic scheme of compound **17, 18, 19, 6, 7**.

Benzyl-β-D-arabinopyranoside (17)[2]

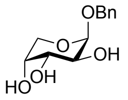

17

To a suspension solution of D-Arabinose **5** (10.0 g, 66.6 mmol) in benzyl alcohol (52 mL, 520 mmol), acetyl chloride (3 mL, 43.8 mmol) was dropwise added with stirring at 0 °C under N_2 atmosphere. The ice-bath was removed after completion of addition and the resulting mixture was heated at 50 °C with vigorous stirring overnight. The reaction mixture became a white suspension and Et_2O (400 mL) was then added to precipitate the benzyl glycoside with stirring at 4 °C. The precipitates were filtered, washed with Et_2O (100 mL) and dried *in vacuo* to give **17** (15.0 g, 94 %) as white powder. R_f 0.35 (MeOH/ $CHCl_3$= 1/9); $[\alpha]^{20}_D$ -208.8 (c 2.0, MeOH); ^{1}H NMR (CD_3OD, 400 MHz) δ 7.41-7.39 (m, 2H), 7.35-7.31 (m, 2H), 7.29-7.25 (m, 1H), 4.88 (d, J = 1.8 Hz, 1H), 4.72 (d, J = 12.0 Hz, 1H), 4.54 (d, J = 12.0 Hz, 1H), 3.87-3.77 (m, 4H), 3.60 (dd, J = 12.8, 2.5 Hz); ^{13}C NMR (CD_3OD, 100 MHz) δ 139.3, 129.5, 129.3, 128.9, 100.2, 71.0, 70.9, 70.6, 70.6, 64.5; HRMS (ESI), Calcd for $C_{12}H_{16}O_5Na$ $[M+Na]^+$ 263.0890; found 263.0891.

Benzyl 4-*O*-acetyl-β-D-arabinopyranoside (18)[2]

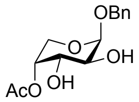

18

To a suspending solution of benzyl glycoside **17** (10.0 g, 41.6 mmol) and catalytic amount of camphor sulfonic acid (CSA, 290 mg, 1.25 mmol) in anhydrous CH_2Cl_2 (200 mL) was dropwise added triethyl orthoacetate (15.3 mL, 83.2 mmol) with stirring at 0 °C under N_2 atmosphere. While completion of addition, the resulting mixture was gradually warmed to room temperature with stirring for another 2 h. The reaction mixture eventually turned to a transparent solution and was quenched by adding triethylamine (NEt_3, 0.6 mL, 4.2 mmol). The neutralized mixture was under reduced pressure to provide a dry residue, which was further dissolved in 90% aqueous AcOH solution (100 mL) and heated to 40 °C for 30 minutes. The reaction mixture was cooled to room temperature and was extracted EtOAc and ice water for 2 times. The combined organic layer was then washed with a saturated aqueous $NaHCO_3$ and brine, dried over anhydrous $MgSO_4$, flitered and concentrated under reduced pressure. The dry residue was purified by recrystallization in $CHCl_3$/hexanes (3 mL/ 350 mL) to give the pure compound **18** (11.2 g, 95 %) as white powder. R_f 0.13 (EtOAc/hexanes = 1/1); $[\alpha]^{20}_D$ -190.0 (c 12.8, MeOH); ^{1}H NMR ($CDCl_3$, 400 MHz) δ 7.37-7.28 (m, 5H), 5.12 (m, 1H), 4.99 (d, J = 3.8 Hz, 1H), 4.74 (d, J = 12.1 Hz, 1H), 4.53 (d, J = 12.1 Hz, 1H), 3.96 (dd, J = 10.2, 3.8 Hz, 1H), 3.85-3.82 (m, 2H), 3.72 (dd, J = 13.1, 2.5 Hz, 1H), 3.06 (br, 1H), 2.63 (br, 1H); ^{13}C NMR ($CDCl_3$, 100 MHz) δ 171.2 , 137.0, 128.7, 128.3, 128.2, 98.1, 71.6, 70.0, 69.9, 68.9, 61.1, 21.2; HRMS (ESI), Calcd for $C_{14}H_{18}O_6Na$ $[M+Na]^+$ 305.0996; found 305.0987.

Benzyl 2,3-*O*-isopropylidene-β-D-arabinopyanoside (19)[2]

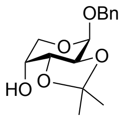

19

To a solution of diol **18** (7.0 g, 24.8 mmol) and catalytic amount of CSA (46 mg, 0.20 mmol) in anhydrous CH_2Cl_2 (90 mL) was slowly added 2-methoxypropene (8.3 mL, 86.8 mmol) with stirring at 0 °C under N_2 atmosphere. The reaction was allowed to warm to room temperature and stirred for further 30 minutes, and then the reaction was quenched by the addition of NEt_3 (about 0.1 mL). The neutralized mixture was concentrated under reduced pressure to give yellow crude oil, which was further dissolved in anhydrous MeOH (105 mL) and followed by adding NaOMe (a 5.4 M solution in MeOH, about 0.13 mL). After stirring for 40 minutes at room temperature under N_2 atmosphere, the reaction mixture was evaporated and the resulting residue was washed with ice and brine and extracted with EtOAc. The organic layer was collected, dried over anhydrous $MgSO_4$, filtered, and concentrated under reduced pressure. The dry residue was purified by column chromatography (EtOAc/hexanes = 3/10) to afford compound **10** (6.2 g, 89 %) as pale-yellow syrup. $[\alpha]^{20}_D$ -183.0 (c 1.0, MeOH); R_f 0.38 (EtOAc/hexanes = 1/1); ^{1}H NMR (CD_3OD, 400 MHz) δ 7.38-7.32 (m, 4H), 7.29-7.26 (m, 1H), 5.25 (d, *J* = 1.4 Hz, 1H), 4.77 (d, *J* = 12.1 Hz, 1H), 4.61 (d, *J* = 12.1 Hz, 1H), 4.23 (m, 1H), 4.06-4.00 (m, 2H), 3.75 (d, *J* = 12.7 Hz), 3.65 (d, *J* = 12.7 Hz, 1H), 1.42 (s, 6H); ^{13}C NMR ($CDCl_3$, 100 MHz) δ 137.6, 128.5, 127.8, 127.7, 110.1, 97.9, 73.4, 71.5, 69.9, 68.4, 63.6, 26.9, 26.8; HRMS (ESI), Calcd for $C_{15}H_{20}O_5Na$ $[M+Na]^+$ 303.1203; found 304.02.

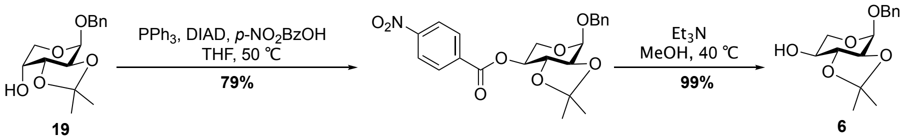

Benzyl 2,3-*O*-isopropylidene-4-*O*-(nitrobenzoyl)-*α*-L-xyloside[2]

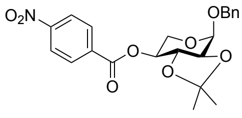

To a vigorous stirring solution of triphenyl phosphine (10.7 g, 38.5 mmol) in anhydrous THF (100 mL) was dropwise added Diisopropyl azodicarboxylate (7.6 mL, 38.5 mmol) at 0 °C under N_2 atmosphere. After stirring for 15 minutes, a solution of compound **10** (7.2 g, 25.7 mmol) in anhydrous THF was slowly added to the mixture, and then stirred for another 15 minutes at same temperature. The solution turned to a viscous, pale-yellow liquid at the end of activation step. To the reaction mixture, one portion of 4-Nitrobenzoic acid (6.4 g, 38.5 mmol) was added by feeding flask with vigorous stirring and the resulting mixture was then heated at 50 °C for overnight. The solvent was removed under reduced pressure to give dry residue that was purified by flash column chromatography (EtOAc/hexanes = 1/4) to give the ester (7.8g, 79 %) as off-white solid. R_f 0.64 (EtOAc/hexanes= 1/3); $[\alpha]^{20}_D$ -34.0 (*c* 1.0, MeOH); ^{1}H NMR (500 MHz, $CDCl_3$) δ 8.28 (d, *J* = 8.9 Hz, 2H), 8.22 (d, *J* = 8.6 Hz, 2H), 7.42–7.34 (m, 4H), 7.34–7.28 (m, 1H), 5.33 (ddd, *J* = 15.8, 10.0, 5.8 Hz, 1H), 5.28 (d, *J* = 2.5 Hz, 1H), 4.82 (d, *J* = 12.1 Hz), 4.68 (d, *J* = 12.1 Hz), 4.34 (t, *J* = 9.7 Hz, 1H), 4.03 (dd, *J* = 10.9, 5.4 Hz, 1H), 3.64 (dd, *J* = 9.5, 2.7 Hz, 1H), 3.51 (t, *J* = 10.5 Hz, 1H), 1.52 (s, CH_3), 1.48 (s, CH_3); ^{13}C NMR (125 MHz, CD_3Cl_3) δ 164.0, 151.0, 137.3, 135.0, 131.3, 128.7, 128.1, 127.8, 123.7, 111.4, 96.6, 76.3, 73.8, 72.9, 70.2, 59.9, 27.0, 26.7; HRMS (ESI) Calcd for $C_{22}H_{23}NO_8$ $[M+Na]^+$ 452.1324, found 453.1316.

Benzyl 2,3-O-isopropylidene-α-L-xylopyranoside (6)[2]

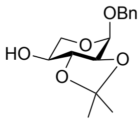

6

Triethylamine (28 mL) was added to ester (62 g, 144.4 mmol) in MeOH (710 mL), and the mixture was stirred at 40 °C for 1h. The solution was concentrated and flash column chromatography on silica gel (EtOAc/hexanes = 1:2) gave compound **6** (40.1 g, 99%) as colourless oil. R*f* 0.27 (EtOAc/hexanes = 1:3); $[\alpha]^{20}_{D}$ -114.6 (*c* 1.0, MeOH); ^{1}H NMR (500 MHz, $CDCl_3$) δ 7.39–7.32 (m, 4H), 7.32–7.26 (m, 1H), 5.19 (d, *J* = 1.4 Hz, 1H), 4.79 (d, *J* = 12.0 Hz), 4.63 (d, *J* = 12.0 Hz), 4.15–3.95 (m, 2H), 3.75 (dd, *J* = 11.9, 4.3 Hz, 1H), 3.45 (d, *J* = 5.7 Hz, 1H), 3.37 (t, *J* = 10.0 Hz, 1H), 2.55 (d, *J* = 2.1 Hz, 1H), 1.47 (s, 3H), 1.44 (s, 3H); ^{13}C NMR (125 MHz, $CDCl_3$) δ 137.5, 128.6, 127.9, 127.8, 111.0, 96.5, 77.39, 76.0, 70.3, 69.8, 63.0, 27.1, 26.7; HRMS (ESI) Calcd for $C_{15}H_{20}O_5$ $[M+Na]^+$ 303.1203, found 304.0536.

Benzyl 4-*C*-Cyano-4-deoxy-2,3-*O*-isopropylidene-β-D-arabinopyanoside (7)[3]

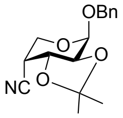

7

Triflouromethanesulfonic anhydride (12.0 mL, 71.3 mmol) was slowly added to a solution of compound **6** (15.0 g, 53.5 mmol) and pyridine (14 mL) in anhydrous CH_2Cl_2 (190 mL) at -10 °C. After the addition was complete, the dry ice-acetone bath was replaced with the ice-water bath and reaction was then allowed to stir an additional 2 h. The reaction mixture was diluted with EtOAc (500 mL), washed successively with satd aq NaCl and ice water. The organic phase was dried over Na_2SO_4 and evaporated under vacuum to give the crude triflate, which was used without further purification. The mixture of the crude triflate, KCN (33.8 g, 519 mmol), 18-crown-6 (15 g), and 4Å molecular sieves (25 g) in DMF (1 L) was stirred at 22 °C for 16 h. The reaction mixture was diluted with EtOAc (400 mL), washed with satd aq NaCl and water, dried over Na_2SO_4 and concentrated. The residue was purified by flash column chromatography on silica gel with a linear gradient of EtOAc/hexanes (from 1/9 to 1/4) to afford compound **7** (12.1 g, 78%) as a pale yellow syrup. R*f* 0.47 (EtOAc/hexanes = 1/3); $[\alpha]^{20}_{D}$ -197.7 (*c* 1.0, MeOH); ^{1}H NMR (500 MHz, $CDCl_3$) δ 7.38–7.33 (m, 4 H), 7.33–7.28 (m, 1H), 5.35 (d, *J* = 2.4 Hz, 1H), 4.76 (d, *J* = 12.1 Hz, 1H), 4.67 (d, *J* = 12.2 Hz, 1H), 4.11 (dd, *J* = 9.6, 4.8 Hz, 1H), 3.92 (d, *J* = 13.3 Hz, 1H), 3.89 (dd, *J* = 9.6, 2.9 Hz, 1H), 3.78 (dd, *J* = 12.0, 2.4 Hz, 1H), 3.32–3.27 (m, 1H), 1.50 (s, 3H), 1.49 (s, 3H); ^{13}C NMR (125 MHz, $CDCl_3$) δ 137.1, 128.7, 128.2, 127.9, 117.2, 111.2, 97.9, 74.8, 70.5, 69.7, 59.5, 34.4, 26.8; HRMS (ESI) Calcd for $C_{16}H_{19}NO_4$ $[M+Na]^+$ 312.1206, found 313.0836.

General hydrogenation set-up in Flow

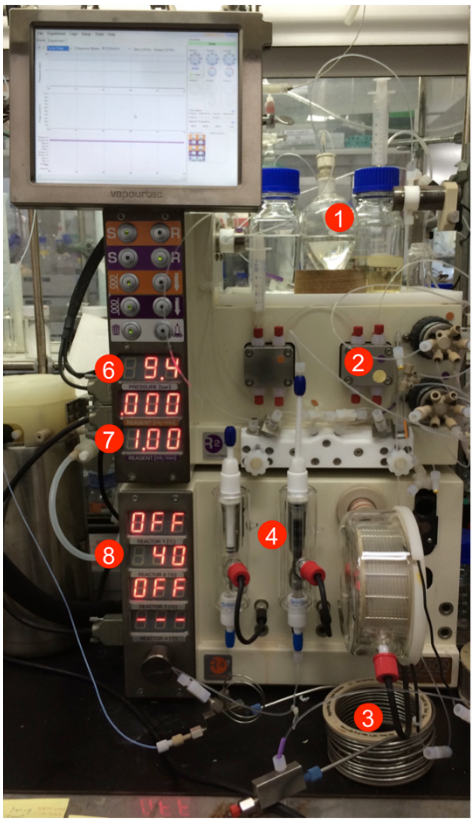

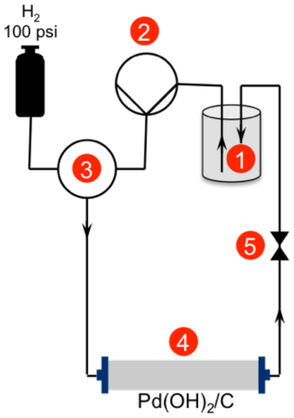

1. Reagents/Solvent
2. Pump
3. Gas/Liquid Reactor
4. Column Reactor
5. Back Pressure Regulator
6. System Pressure
7. Flow Rate
8. Temperature

A Vapourtec R2C^{+}/R4 was equipped with the following device: Polytetrafluoroethane (PTFE) tubing (i.d. 1 mm, 10 mL), gas/liquid reactor, and column reactor packed. The gas/liquid reactor was applied to sufficiently mix solution and H_2, held at 100 psi by a variable regulator. The system pressure was maintained at 10 bar to avoid out-gassing. The column reactor was packed by 2 grams $Pd(OH)_2/C$ and Celite (1/1 W/W), primed by MeOH before the continuous flow reaction and maintained at certain temperatures during the reaction. The starting material in MeOH was passed through the continuous flow system and ran a cycle.

Chemical synthesis and characterization of compound 8–13.[3]

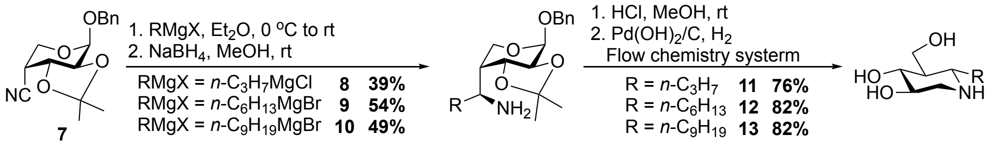

Scheme S2. Synthetic scheme of compound **11-13**.

Benzyl 4-[(*S*)-1-aminobutyl]-4-deoxy-2,3-*O*-isopropylidene-α-L-xylopyranoside (8)[3]

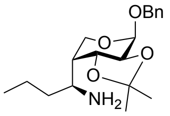

8

n-C_3H_7MgCl (23.0 mL of 2.0 M solution in Et_2O) was added dropwise by addition funnel to a solution of compound **7** (2.2 g, 7.6 mmol) in anhydrous Et_2O (383.7 mL) at 0 °C under an atmosphere of nitrogen, and then slowly warmed to 20 °C and stirred for 36 h. The reaction mixture was cooled to 0 °C, $NaBH_4$ (1.7 g, 44.9 mmol) was added in one portion and then anhydrous MeOH (192 mL) was added dropwise by addition funnel. The reaction mixture was warmed to 22 °C after the addition was completed and allowed to stir 24 h. Water (200 mL) was added to the reaction mixture, and then the mixture was diluted with EtOAc (250 mL), washed with satd aq NaCl and water, dried over $MgSO_4$ and concentrated. The residue was purified by flash column chromatography on silica gel (MeOH/$CHCl_3$ = 1/20) to afford compound **8** (983.1mg, 39%) as pale-yellow syrup. R_f 0.30 (MeOH/$CHCl_3$ = 1/20); $[\alpha]^{20}_D$ -159.7 (*c* 0.1, MeOH); ^1H NMR (500 MHz, $CDCl_3$) δ 7.38–7.32 (m, 4H), 7.31–7.26 (m, 1H), 5,24 (d, *J* = 3.3 Hz, 1H), 4.75 (d, *J* = 12.2 Hz, 1H), 4.61 (d, *J* = 12.1 Hz, 1H), 4.32 (dd, *J* = 10.0, 4.8 Hz, 1H), 3.87 (dd, *J* = 10.0 Hz, 3.3 Hz, 1H), 3.73 (d, *J* = 12.6 Hz, 1H), 3.67 (dd, *J* = 12.6, 2.8 Hz), 3.29–3.23 (m, 1H,), 2.09–2.04 (m, 1H), 1.64–1.57 (m, 1H), 1.55–1.47 (m, 1H), 1.46 (s, 6H), 1.40-1.20 (m, 2H), 0.95 (t, *J* = 7.2 Hz, 3H); ^{13}C NMR (125 MHz, $CDCl_3$) δ 137.7, 128.5, 127.8, 127.7, 109.3, 97.8, 74.5, 73.0, 69.7, 60.0, 48.2, 47.3, 37.5, 27.1, 26.5, 18.7, 14.4; HRMS (ESI) Calcd for $C_{19}H_{29}NO_4$ $[M+H]^+$ 336.2169, found 336.2161.

Benzyl 4-[(*S*)-1-aminoheptyl]-4-deoxy-2,3-*O*-isopropylidene-α-L-xylopyranoside (9)[3]

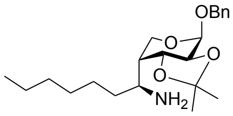

9

n-$C_6H_{13}MgBr$ (16.0 mL of 2.0 M solution in Et_2O) was added dropwise by addition funnel to a solution of compound **7** (3.0 g, 10.4 mmol) in anhydrous Et_2O (420 mL) at room temperature under an atmosphere of nitrogen, and stirred for 30 h. The reaction mixture was cooled to 0 °C, $NaBH_4$ (2.4 g, 63.4 mmol) was added in one portion and then anhydrous MeOH (120 mL) was added dropwise by addition funnel. After the addition was completed, the ice-water bath was removed and the reaction mixture was allowed to stir at 20 °C for 36 h. water (100 mL) and Et_2O (100 mL) was added to the reaction mixture. White solid was filtered and washed with Et_2O (30 mL). The combines filtrates were washed with water, dried over $MgSO_4$, and concentrated. The residue was purified by flash column chromatography on silica gel (MeOH/$CHCl_3$ = 1/19) gave compound **9** (2.1 g, 54%) as pale-yellow syrup. R_f 0.17 (MeOH/$CHCl_3$ = 1/19); $[\alpha]^{20}_D$ -140.2 (*c* 0.02, CH_2Cl_2); ^1H NMR (500 MHz, $CDCl_3$) δ 7.36–7.32 (m, 4H), 7.29–7.28 (m, 1H), 5.24 (d, *J* = 3.2 Hz, 1H), 4.75 (d, *J* = 12.2 Hz, 1H), 4.61 (d, *J* = 12.2 Hz, 1H), 4.32 (dd, *J* = 10.0, 4.8 Hz, 1H), 3.86 (dd, *J* = 10.0, 3.3 Hz, 1H), 3.72 (d, *J* = 12.5 Hz, 1H), 3.68 (dd, *J* = 12.6, 2.6 Hz, 1H), 3.33–3.29 (m, 3H), 2.17–2.15 (m, 1H), 1.69–1.65 (m, 1H), 1.46 (s, 3H), 1.46 (s, 3H), 1.37–1.30 (m, 9H), 0.90 (t, *J* = 7.2 Hz, 3H); ^{13}C NMR (125 MHz, $CDCl_3$) δ 137.4, 128.2, 127.5, 127.3, 109.0, 97.4, 74.2, 72.6, 69.4, 59.6, 48.0, 46.8, 34.9, 31.7, 29.4, 26.8, 26.2, 25.1, 22.5, 13.9; HRMS (ESI) Calcd for $C_{22}H_{35}NO_4$ $[M+H]^+$ 378.2629, found 378.2639.

Benzyl 4-[(*S*)-1-aminodecyl]-4-deoxy-2,3-*O*-isopropylidene-α-L-xylopyranoside (10)[3]

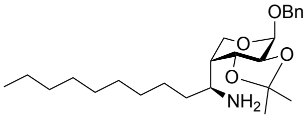

10

n-$C_9H_{19}MgBr$ (10.5 ml of 1.0 M solution in Et_2O) was added dropwise by addition funnel to a solution of compound **7** (1.0 mg, 3.46 mmol) in anhydrous Et_2O (140 mL) at 0 °C under an atmosphere of nitrogen, and then slowly warmed to 20 °C and stirred for 24 h. The reaction mixture was cooled to 0 °C, $NaBH_4$ (770.0 mg, 20.5 mmol) was added in one portion and then anhydrous MeOH (50 mL) was added dropwise by addition funnel. The reaction mixture was warmed to 20 °C after the addition was complete and allowed to stir 20 h. Water (25 mL) was added to the reaction mixture, and then the mixture was diluted with EtOAc (50 mL), washed with brine and water, dried over $MgSO_4$ and concentrated. The residue was purified by flash column chromatography on silica gel (MeOH/$CHCl_3$ = 1/20) to afford compound **10** (710.4 mg, 49%) as pale-yellow syrup. R_f 0.25 (MeOH/$CHCl_3$ = 1/19); $[\alpha]^{20}_D$ -59.7 (*c* 0.01, MeOH); ^1H NMR (500 MHz, $CDCl_3$) δ7.37–7.26 (m, 5H), 5,24 (d, *J* = 3.0 Hz, 1H), 4.75 (d, *J* = 12.1 Hz, 1H), 4.61 (d, *J* = 12.1 Hz, 1H), 4.32 (dd, *J* = 10.0, 4.8 Hz, 1H), 3.87 (dd, *J* = 10.0 Hz, 3.2 Hz, 1H), 3.73 (d, *J* = 12.5 Hz, 1H), 3.67 (dd, *J* = 12.6, 2.5 Hz), 3.26–3.22 (m, 1H), 2.06–2.04 (m, 3H), 1.65–1.59 (m, 1H), 1.46 (s, 6H), 1.26 (br, 15H), 0.88 (t, *J* = 6.8 Hz, 3H); HRMS (ESI) Calcd for $C_{25}H_{41}NO_4$ $[M+H]^+$ 420.3108, found 420.3106.

(3*R*, 4*R*, 5*R*, 6*S*)-6-propyl-5-(hydroxymethyl)piperidine-3,4-diol (11)

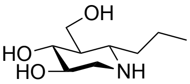

11

A mixture of compound **8** (983.1 mg, 2.93 mmol), HCl (*conc.* 251.5 μL) in MeOH (6 mL) was stirred at 22 °C for 1h and concentrated *in vacuo* to get crude yellow residue. The residue was dissolved in MeOH (2 L), passed through the continuous flow system with the flow rate of 0.5 mL min^{-1} at 40 °C for 66 h. The reaction mixture at the output stream was concentrated *in vacuo* and purified by flash column chromatography on silica gel (MeOH/$CHCl_3$/NH_4OH= 30/69/1) to afford compound **11** (432.5 mg, 78%) as a colorless syrup. R_f 0.42 (MeOH/$CHCl_3$/NH_4OH= 30/65/5); $[\alpha]^{20}_D$ -12.3 (*c* 0.01, MeOH); ^1H NMR (500 MHz, D_2O) δ4.05 (dd, *J* = 12.1, 1.5 Hz, 1H), 3.85 (dd, *J* = 12.1, 2.3 Hz, 1H), 3.81–3.74 (m, 1H), 3.65 (dd, *J* = 10.2, 10.2 Hz, 1H), 3.46 (dd, *J* = 12.4, 5.0 Hz, 1H), 3.22–3.17 (m, 1H), 2.83 (t, *J* = 12.0 Hz, 1H), 1.96–1.88 (m, 1H), 1.69–1.61 (m, 2H), 1.60–1.49 (m, 1H), 1.46–1.35 (m, 1H), 1.02 (t, *J* = 7.3 Hz); ^{13}C NMR (125 MHz, D_2O) δ71.1, 69.5, 56.5, 56.0, 46.9, 45.9, 32.2, 17.5, 13.2; HRMS (ESI) Calcd for $C_9H_{19}NO_3$ $[M+Na]^+$ 190.1438, found 190.1435.

(3*R*,4*R*,5*R*,6*S*)-6-hexyl-5-(hydroxymethyl)piperidine-3,4-diol (12)

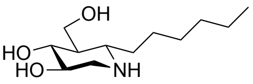

12

A mixture of compound **9** (660.0 mg, 1.75 mmol), HCl (*conc.* 50.0 μL) in MeOH (100 mL) was stirred at 22 °C for 1h and concentrated i*n vacuo* to get crude yellow residue. The residue was dissolved in MeOH (600 mL), passed through the continuous flow system with the flow rate of 1.0 mL min^{-1} at 40 °C for 30 h. The reaction mixture at the output stream was concentrated *in vacuo* and purified by flash column chromatography on silica gel (MeOH/$CHCl_3$= 1/4) to afford compound **12** (331.5 mg, 82%) as a colorless syrup. R_f 0.47 (MeOH/$CHCl_3$ = 1/4); $[\alpha]^{20}_D$ -5.0 (*c* 0.002, MeOH); ^1H NMR (500MHz, D_2O) δ 3.93 (dd, *J* = 11.9, 2.3 Hz, 1H), 3.78 (dd, *J* = 11.8, 2.7 Hz, 1H), 3.51 (ddd, *J* = 14.0, 9.1, 4.9 Hz,1H), 3.48 (dd, *J* = 19.3, 9.2 Hz, 1H), 3.13 (dd, *J* = 11.8, 4.6 Hz, 1H), 2.65 (ddd, *J* = 8.3, 8.3, 2.8 Hz,1H), 2.44 (dd, *J* = 11.2, 10.6 Hz, 1H), 1.75–1.71 (m, 1H), 1.41–1.37 (m, 10H), 0.89 (t, *J* = 6.3 Hz, 3H); ^{13}C NMR (125 MHz, D_2O) δ 72.9, 72.1, 57.4, 54.9, 49.0, 48.0, 31.5, 30.9, 28.6, 24.3, 22.0, 13.4; HRMS (ESI) Calcd for $C_{12}H_{25}NO_3$ $[M+H]^+$ 232.1911, found 232.1907.

(3*R*, 4*R*, 5*R*, 6*S*)- 6-nonyl-5-(hydroxymethyl)piperidine-3,4-diol (13)

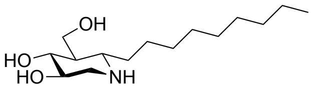

13

A mixture of compound **10** (448.0 mg, 1.07 mmol), HCl (*conc.* 49.6 μL) in MeOH (100 mL) was stirred at 22 °C for 1 h and concentrated *in vacuo* to get crude yellow residue. The residue was dissolved in MeOH (900 mL), passed through the continuous flow system with the flow rate of 1.0 mL min^{-1} at 40 °C for 30 h. The reaction mixture at the output stream was concentrated *in vacuo* and purified by flash column chromatography on silica gel (MeOH/CHCl$_3$= 3/7) to afford compound **13** (239.4 mg, 82%) as a colorless syrup. R$_f$ 0.60 (MeOH/CHCl$_3$/NH$_4$OH = 30/65/5); $[\alpha]^{20}_D$ -12.2 (*c* 0.005, MeOH); ^{1}H NMR (500 MHz, D$_2$O) δ 3.99 (d, *J* = 10.3, 1H), 3.75 (d, *J* = 10.3Hz, 1H), 3.68 (td, *J* = 10.0, 5.0 Hz, 1H), 3.57 (t, *J* = 9.8 Hz, 1H), 3.36 (dd, *J* = 12.3, 4.6 Hz, 1H), 3.03 (t, *J* = 7.7 Hz, 1H), 2.71 (t, *J* = 11.7 Hz, 1H), 1.88–1.83 (m, 1H), 1.57-1.31 (m, 16H), 0.89 (t, *J* = 13.0 Hz, 3H); ^{13}C NMR (125 MHz, D$_2$O) δ 71.6, 70.0, 56.8, 56.0, 47.5, 46.3, 31.5 , 30.6, 29.2, 29.1, 28.9, 24.3, 22.3, 13.6; HRMS (ESI) Calcd for $C_{15}H_{31}NO_3$ [M+Na]$^+$ 274.2377, found 274.2374.

Chemical synthesis and characterization of compound 14–16, 20–21 and 2–4.

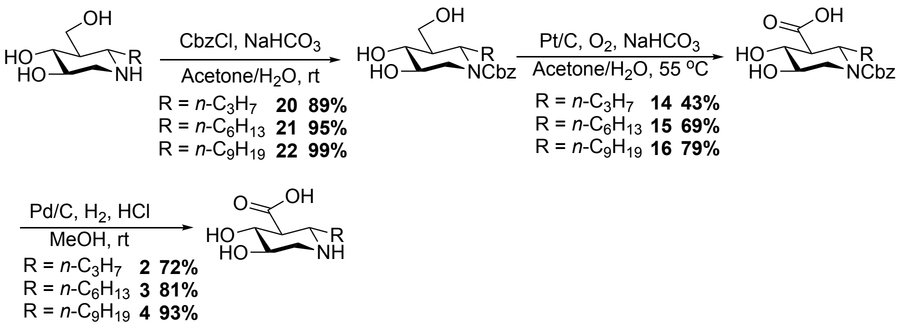

Scheme S3. Synthetic scheme of compound **2-4**.

(2*S*,3*R*,4*R*,5*R*)-benzyl 4,5-dihydroxy-3-(hydroxymethyl)-2-propylpiperidine-1-carboxylate (20)

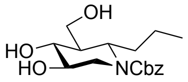

20

NaHCO$_3$ (449.2 mg, 5.35 mmol) and CbzCl (694.0 μL, 4.86 mmol) were added to compound **11** (460.0 mg, 2.43 mmol) in acetone/H$_2$O (48.6 mL; v/v 7/3), and stirred at ambient temperature for 1.5 h. The solution was concentrated and purified by flash chromatography on silica gel (MeOH/CHCl$_3$ = 5/95) gave compound **20** (699.5 mg, 89%) as colourless oil. R$_f$ 0.39 (MeOH/CHCl$_3$ = 1/9); $[\alpha]^{20}_D$ +19.0 (*c* 0.01, MeOH); ^{1}H NMR (400 MHz, CDCl$_3$) δ 7.35–7.28 (m, 5H), 5.15 (d, *J* = 12.6 Hz, 1H), 5.11 (d, *J* = 12.6 Hz, 1H), 4.13–4.10 (m, 1H), 3.94 (dd, *J* = 14.3, 1.6 Hz, 1H), 3.71–3.67 (m, 4H), 3.38 (dd, *J* = 14.3, 3.0 Hz, 1H), 2.25 (br, 1H), 1.90–1.81 (m, 3H), 1.67–1.64 (m, 1H), 1.56–1.47(m, 2H), 0.88 (t, *J* = 7.3 Hz, 3H); ^{13}C NMR (100 MHz, CDCl$_3$) δ 157.1, 136.6, 128.4, 128.0, 127.6, 72.4, 70.0, 67.4, 64.3, 52.6, 45.1, 42.1, 35.3, 19.5, 13.8; HRMS (ESI) Calcd for $C_{17}H_{25}NO_5$ [M+Na]$^+$ 346.1625, found 346.1627.

(2*S*,3*S*,4*R*,5*R*)-1-((benzyloxy)carbonyl)-4,5-dihydroxy-2-propylpiperidine-3-carboxylic acid (14)

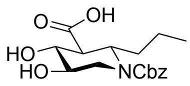

14

To a solution of compound **20** (66.1 mg, 0.20 mmol), sodium bicarbonate (132.2 mg, 1.57 mmol) and Pt/C (10%, 132.2 mg) in H_2O/acetone/IPA (132 mL, v/v/v 6/5/1) was bubbled with oxygen at 55 °C for 24 h. The solution was filtered by Celite, concentrated *in vacuo* and purified by column chromatography on silica gel ($MeOH/CHCl_3/NH_3$= 2.5/7.3/0.2) to afford compound **14** (48.3 mg, 43%) as colourless oil. R_f 0.36 ($MeOH/CHCl_3/NH_4OH$ = 2.8/7/0.2); $[\alpha]^{20}_D$ +5.8 (*c* 0.01, MeOH); ^1H NMR (400 MHz, D_2O) δ 7.59–7.54 (m, 5H), 5.30 (s, 2H), 4.45 (br, 1H), 4.03–3.97 (m, 2H), 3.86 (br, 1H), 3.55 (dd, *J* = 14.8, 3.2 Hz, 1H), 2.58 (t, 1H, *J* = 13.0 Hz, 1H), 1.91 (td, *J* = 14.6, 8.1 Hz, 1H), 1.69 (td, *J* = 13.9, 7.2 Hz, 1H), 1.40–1.34 (m, 2H), 0.97 (t, *J* = 7.2 Hz, 3H); ^{13}C NMR (100 MHz, D_2O) δ 180.1, 157.5, 136.4, 128.6, 128.2, 127.6, 71.1, 71.0, 67.4, 54.6, 53.4, 43.5, 34.7, 18.5, 13.0; HRMS (ESI) Calcd for $C_{17}H_{23}NO_6$ $[M+Na]^+$ 360.1418, found 360.1419.

(2S,3S,4R,5R)-4,5-dihydroxy-2-propylpiperidine-3-carboxylic acid (2)

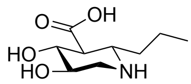

2

The compound **14** (5.6 mg, 0.02 mmol) was subjected to hydrogenolysis by stirring with Pd/C (10%, 11.2 mg) and HCl (*conc.* 3 μL) in EtOH (16.6 mL) for 2 h at 22 °C under an atmosphere of hydrogen. The mixture was filtered through Celite, concentrated *in vacuo*, and purified by flash column chromatography on silica gel (ACN/H_2O = 1/3). The compound 2 was colorless oil (2.4 mg, 72%). R_f 0.38 (ACN/H_2O = 3/7); $[\alpha]^{20}_D$ -2.1 (*c* 0.01, MeOH); ^1H NMR (500 MHz, D_2O) δ 3.73–3.69 (m, 2H), 3.45 (dd, *J* = 12.5, 3.9 Hz, 1H), 3.24–3.19 (m, 1H), 2.91–2.84 (m, 1H), 2.40–2.33 (m, 1H), 1.70–1.55 (m, 2H), 1.54–1.45 (m, 1H); 1.41–1.31 (m, 1H), 0.93 (t, *J* = 7.3 Hz, 3H); ^{13}C NMR (125 MHz, D_2O) δ 176.8, 73.8, 68.4, 57.4, 56.3, 46.6, 33.4, 17.8, 13.0; HRMS (ESI) Calcd for $C_9H_{17}NO_4$ $[M+H]^+$ 204.1230, found 204.1231.

(2*S*,3*R*,4*R*,5*R*)-benzyl 2-hexyl-4,5-dihydroxy-3-(hydroxymethyl)piperidine-1-carboxylate (21)

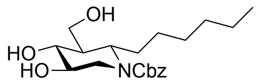

21

$NaHCO_3$ (407 mg, 4.84 mmol) and CbzCl (138 μL, 0.97 mmol) were added to compound **12** (112 mg, 0.48 mmol) in acetone/H_2O (20 mL, v/v 14/6), and the reaction mixture was stirred at 22 °C for 2 h. The solution was concentrated and purified by flash chromatography on silica gel ($MeOH/CHCl_3$ = 1/9) to gave compound **21** (186 mg, 95%) as colourless oil. R_f 0.38 ($MeOH/CHCl_3$ = 1/9); $[\alpha]^{25}_D$ +24.0 (*c* 0.01, CH_2Cl_2); ^1H NMR (500MHz, MeOD) δ 7.38–7.27 (m, 5 H), 5.16 (d, *J* = 12.4 Hz, 1H), 5.10 (d, *J* = 12.2 Hz, 1H), 4.32–4.29 (m, 1H), 3.94 (dd, *J* = 14.4, 2.6 Hz, 1H), 3.68 (dd, *J* = 10.8, 7.3 Hz, 1H), 3.67–3.64 (m, 2H), 3.60 (dd, *J* = 11.0, 4.9 Hz, 1H), 3.36 (dd, *J* = 14.1, 1.8 Hz, 1H), 2.02–1.94 (m, 1H), 1.75–1.74 (m, 1H), 1.60–1.55 (m, 1H), 1.34–1.23 (m, 8H), 0.87 (t, *J* = 6.7 Hz, 3H); ^{13}C NMR (125 MHz, MeOD) δ 158.5, 138.4, 129.4, 128.9, 128.7, 71.6, 70.3, 68.1, 63.9, 53.4, 47.3, 42.4, 34.0, 33.0, 30.2, 27.6, 23.6, 14.4; HRMS (ESI) Calcd for $C_{20}H_{31}NO_5$ $[M+Na]^+$ 388.2098, found 388.2094.

(2*S*,3*S*,4*R*,5*R*)-1-((benzyloxy)carbonyl)-4,5-dihydroxy-2-hexylpiperidine-3-carboxylic acid (15)

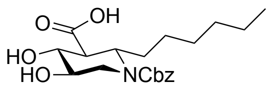

15

To a solution of compound **21** (50 mg, 0.14 mmol), sodium bicarbonate (100 mg, 1.19 mmol) and Pt/C (10%, 100 mg) in H_2O/acetone/IPA (132 mL, v/v/v 6/5/1) was bubbled with oxygen at 55 °C for 24 h. The solution was filtered by Celite, concentrated *in vacuo* and purified by column chromatography on silica gel ($MeOH/CHCl_3/NH_3$= 2.5/7.3/0.2) to afford compound **14** (36 mg, 69%) as colourless oil. R_f 0.38 ($MeOH/CHCl_3$/ NH_4OH = 2.8/7/0.2); $[\alpha]^{20}_D$ +5.8 (*c* 0.01, MeOH); ^1H NMR (400 MHz,

D_2O) δ 7.59–7.54 (m, 5H), 5.30 (s, 2H), 4.45 (br, 1H), 4.03–3.97 (m, 2H), 3.86 (br, 1H), 3.55 (dd, J = 14.8, 3.2 Hz, 1H), 2.58 (t, 1H, J = 13.0 Hz, 1H), 1.91 (td, J = 14.6, 8.1 Hz, 1H), 1.69 (td, J = 13.9, 7.2 Hz, 1H), 1.40–1.34 (m, 2H), 0.97 (t, J = 7.2 Hz, 3H); ^{13}C NMR (100 MHz, D_2O) δ 180.1, 157.5, 136.4, 128.6, 128.2, 127.6, 71.1, 71.0, 67.4, 54.6, 53.4, 43.5, 34.7, 18.5, 13.0; HRMS (ESI) Calcd for $C_{17}H_{23}NO_6$ $[M+Na]^+$ 360.1418, found 360.1419.

(2*S*,3*S*,4*R*,5*R*)-2-hexyl-4,5-dihydroxypiperidine-3-carboxylic acid (3)

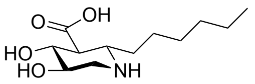

3

A mixture of compound **15** (20 mg, 0.05 mmol), Pd/C (10%, 40 mg), and HCl (*conc.* 50 μL) in MeOH (20 mL) was stirred under an atmosphere of hydrogen at 20 °C for 3 h. The reaction mixture was filtered through a pad of Celite, the filter cake was washed with MeOH and the filtrates were concentrated. The residue was purified by flash column chromatography on silica gel (IPA/H_2O/NH_4OH = 8/1/1) to afford compound **3** (10.5 mg, 81%) as a colorless syrup. R_f 0.4 (IPA/H_2O/NH_4OH = 8/1/1); $[\alpha]^{25}_D$ -5.3 (*c* 0.0053, MeOH); ^1H NMR (500MHz, D_2O) δ 3.80–3.69 (m, 2H), 3.52 (dd, J = 12.3, 4.4 Hz, 1H), 3.30 (ddd, J = 11.5, 7.8, 3.9 Hz, 1H), 2.94 (dd, J = 12.3, 11.2 Hz, 1H), 2.42 (dd, J = 10.6, 10.6 Hz, 1H), 1.75–1.71 (m, 1H), 1.67–1.61 (m, 1H), 1.50–1.47 (m, 1H), 1.35–1.30 (m, 8H), 0.88 (t, J = 6.8 Hz, 3H); ^{13}C NMR (125 MHz, D_2O) δ 176.2, 73.5, 67.9, 57.7, 55.7, 46.3, 30.9, 30.5, 28.0, 24.0, 21.8, 13.3; HRMS (ESI) Calcd for $C_{12}H_{23}NO_4$ $[M+H]^+$ 246.1700, found 246.1700.

Benzyl (2*S*,3*R*,4*R*,5*R*)-4,5-dihydroxy-3-(hydroxymethyl)-2-nonylpiperidine-1-carboxylate (22)

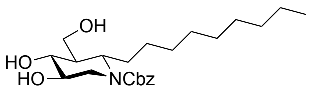

22

$NaHCO_3$ (350.3 mg, 4.17 mmol) and CbzCl (119 μL, 0.83 mmol) were added to compound **13** (113 mg, 0.42 mmol) in acetone/H_2O (18 mL, v/v 12/6), and the reaction mixture stirred at 22 °C (3 h). The solution was concentrated and flash column chromatography on silica gel (MeOH/$CHCl_3$ = 1/9) gave the compound **22** as colorless oil (167.8 mg, 99%). R_f 0.25 (MeOH/$CHCl_3$ = 1/19); $[\alpha]^{25}_D$ +24.0 (*c* 0.01, CH_2Cl_2); ^1H NMR (500 MHz, MeOD) δ 7.37 (d, *J* = 7.2Hz, 2 H), 7.33 (t, *J* = 7.0Hz, 2 H), 7.27 (d, *J* = 7.1Hz, 1 H), 5.13 (ABq, 2 H), 4.31–4.28 (m, 1 H), 3.93 (dd, *J* = 14.0 and 2.2 Hz, 1 H), 3.68 (dd, *J* = 11.0 and 7.2 Hz, 1 H), 3.68–3.66 (m, 2 H), 3.61 (dd, *J* = 10.9 and 4.9 Hz, 1 H), 3.37 (dd, *J* = 14.2 and 2.0 Hz, 1 H), 1.97 (ddd, *J* = 18.2 and 9.6 and 4.6 Hz, 1 H), 1.75–1.74 (m, 1 H), 1.58 (ddd, *J* = 19.0 and 9.6 and 4.2 Hz, 1 H), 1.33–1.18 (m, 14 H), 0.87 (t, *J* = 6.8 Hz, 3 H); ^{13}C NMR (125 MHz, MeOD) δ 158.5, 138.4, 129.4, 128.9, 128.7, 71.7, 70.4, 68.1, 64.0, 53.5, 47.3, 42.6, 34.0, 33.0, 30.7, 30.6, 30.5, 30.4, 27.6, 23.7, 14.4; HRMS (ESI) Calcd for $C_{23}H_{37}NO_5$ $[M+Na]^+$ 430.2562, found 430.2564.

(2*S*,3*S*,4*R*,5*R*)-4,5-dihydroxy-2-nonylpiperidine-3-carboxylic acid (4)

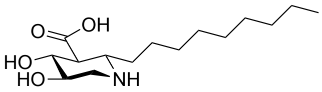

4

A mixture of compound **22** (38 mg, 0.09 mmol), Pt/C (10%, 80 mg), and $NaHCO_3$ (90 mg) in H_2O/acetone/IPA (120 mL, v/v/v 60/40/20) was bubbled with oxygen through the suspension at 55 °C for 50 h. After the solution was concentrated, the residue was diluted with EtOAc and washed with *sat.* $NH_4Cl_{(aq)}$ and brine, dried over $MgSO_4$, filtered, and concentrated to afford crude product **16** (31 mg) as a white solid.

A mixture of crude compound **16** (31 mg, 0.07 mmol), Pd/C (10%, 80 mg), and HCl (*conc.* 400 μL) in MeOH (20 mL) was stirred under an atmosphere of hydrogen at 22 °C for 2 h. The reaction mixture was filtered through a pad of Celite and the filter cake was washed with MeOH. The filtrates were concentrated. The residue was purified by flash chromatography (IPA/H_2O/NH_4OH = 8/1/1) to afford compound **4** (19.6 mg, 73% in 2 steps) as a colorless syrup. R_f 0.37 (IPA/H_2O/NH_4OH = 8/1/1); $[\alpha]^{20}_D$ 10.8 (*c* 0.004, MeOH); ^1H NMR (500 MHz, D_2O) δ 3.63–3.59 (m, 2H), 3.36 (dd, *J* = 12.9, 3.3 Hz, 1H), 3.13–3.08 (m, 1H), 2.78 (dd, *J* = 12.6, 11.4 Hz, 1H), 2.42 (dd, *J* = 10.2, 10.2 Hz, 1H), 1.63–1.58 (m, 1H), 1.53–1.47 (m, 1H), 1.41–1.35 (m, 1H), 1.25–1.19 (m, 8H), 0.78 (t, *J* = 6.2 Hz, 3H); ^{13}C NMR (125 MHz, D_2O) δ 175.3, 73.3, 67.8, 57.5, 54.7, 46.3, 31.2, 30.9, 28.6, 28.4, 28.3, 28.2, 24.0, 22.0, 13.4; HRMS (ESI) Calcd for $C_{12}H_{23}NO_4$ $[M+H]^+$ 288.2168, found 288.2169.

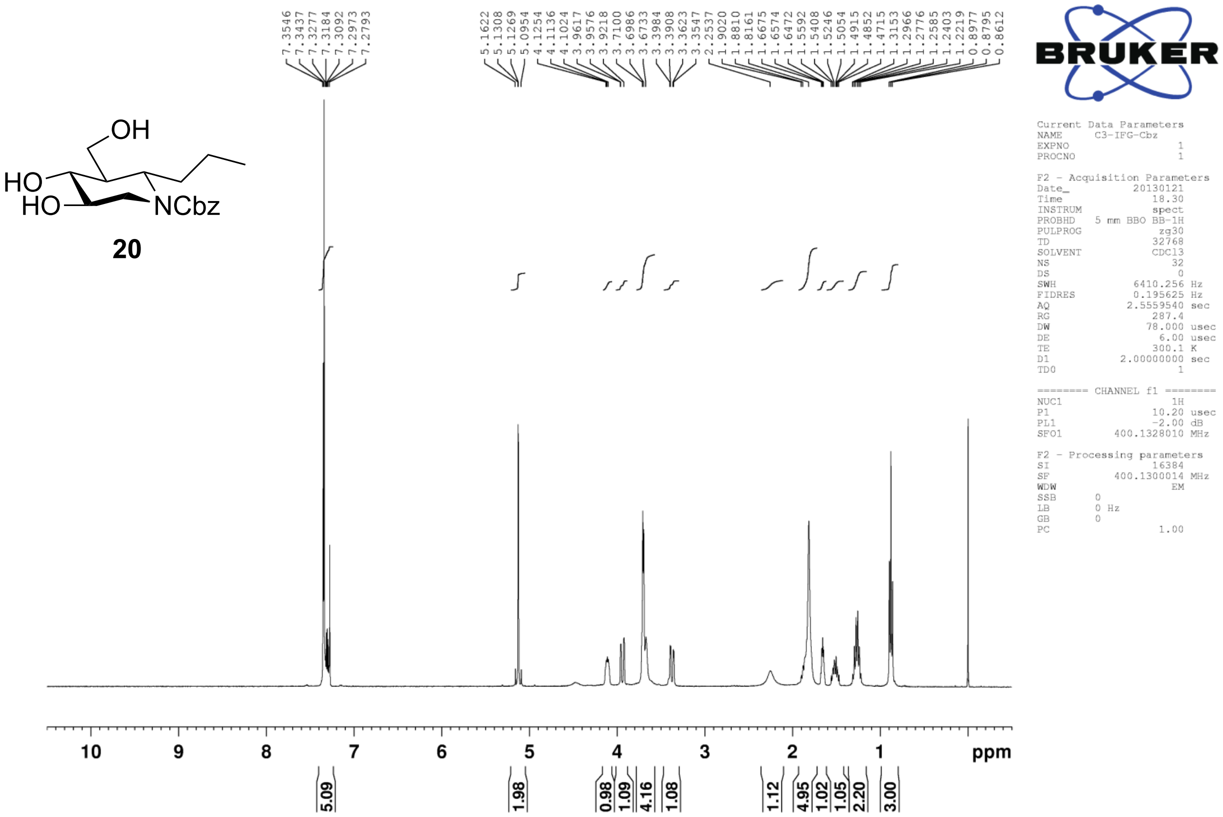

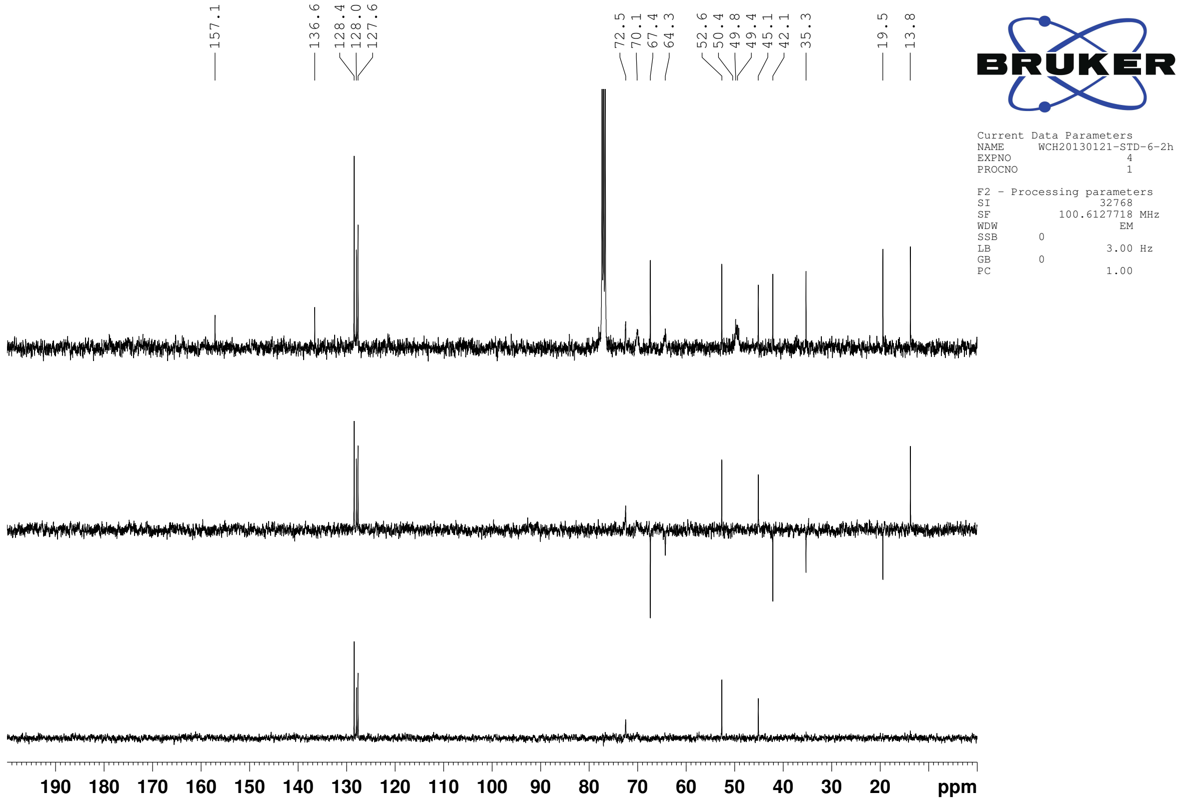

^{1}H-NMR spectrum ($CDCl_3$, 400 MHz) and ^{13}C-NMR spectrum ($CDCl_3$, 100 MHz) of **20**.

2

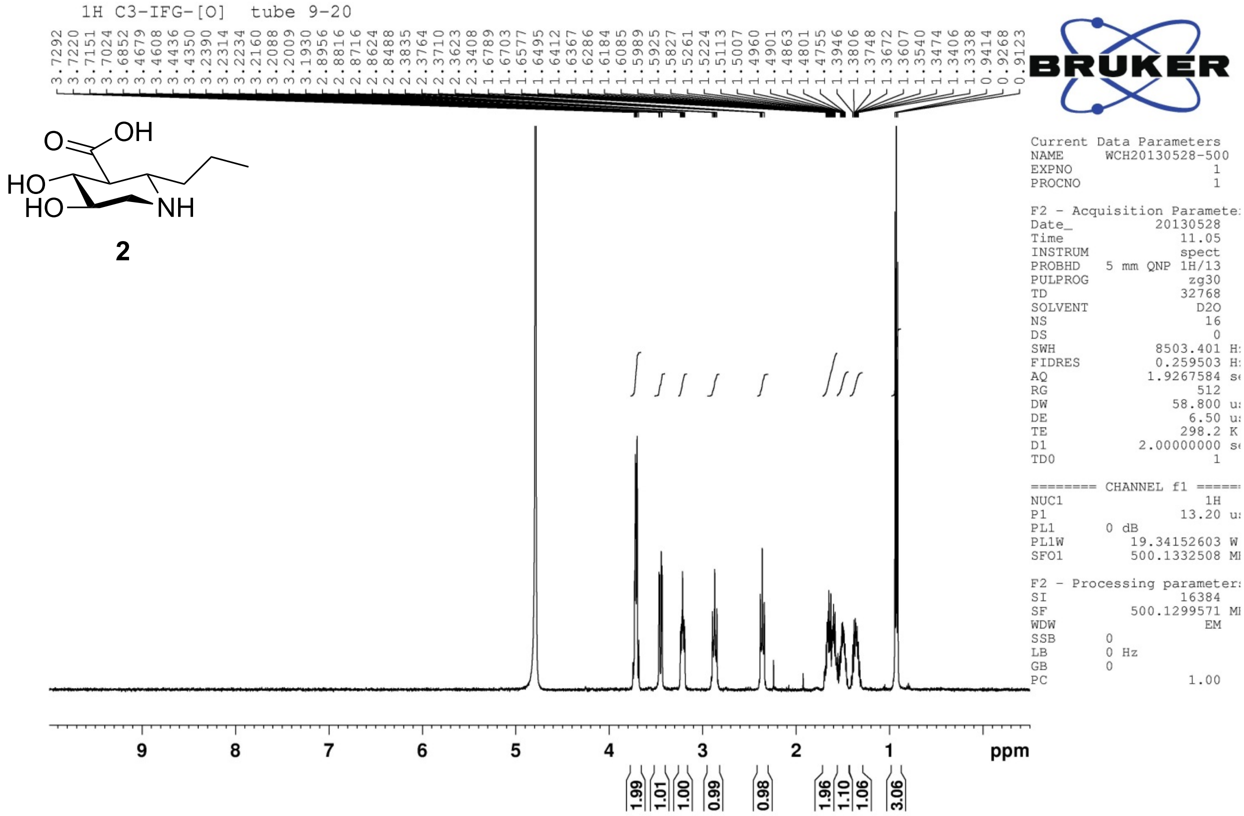

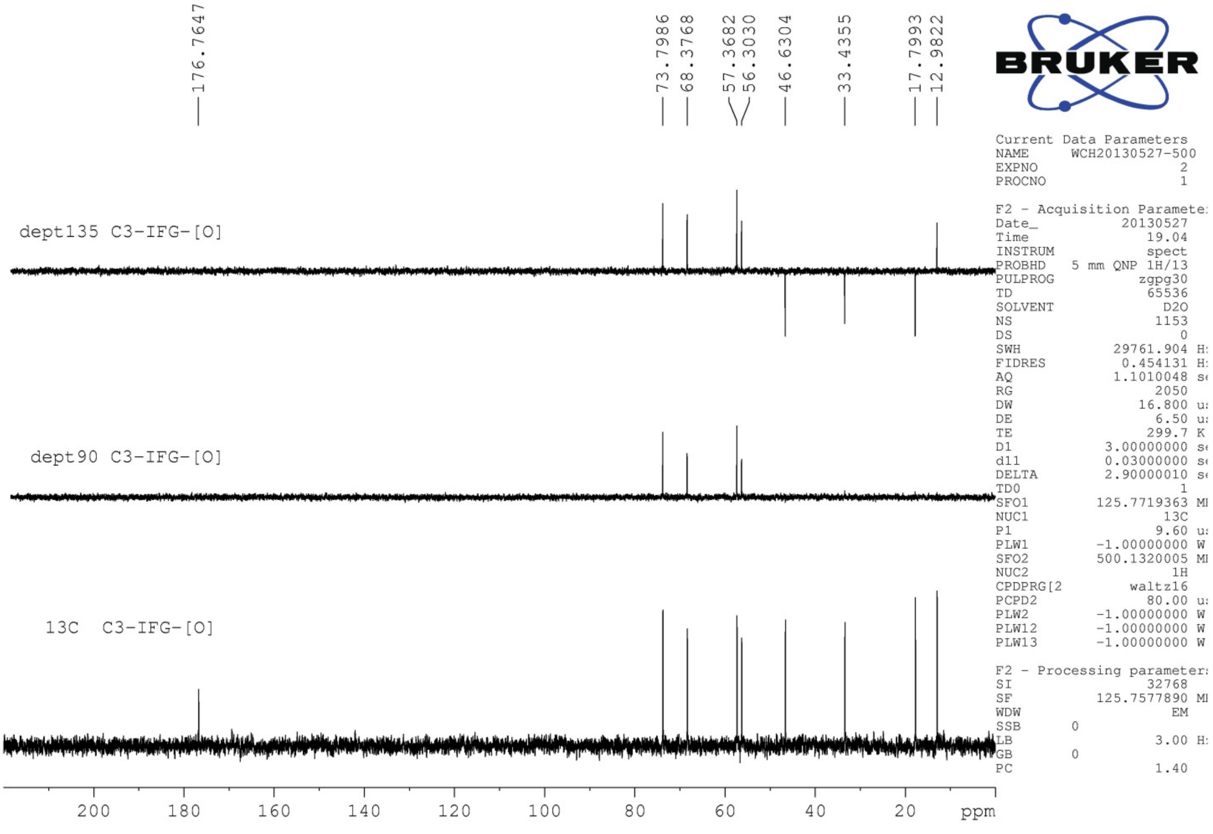

^{1}H-NMR spectrum ($CDCl_3$, 500 MHz) and ^{13}C-NMR spectrum ($CDCl_3$, 125 MHz) of **2**.

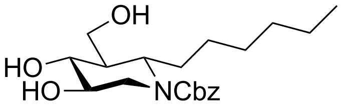

21

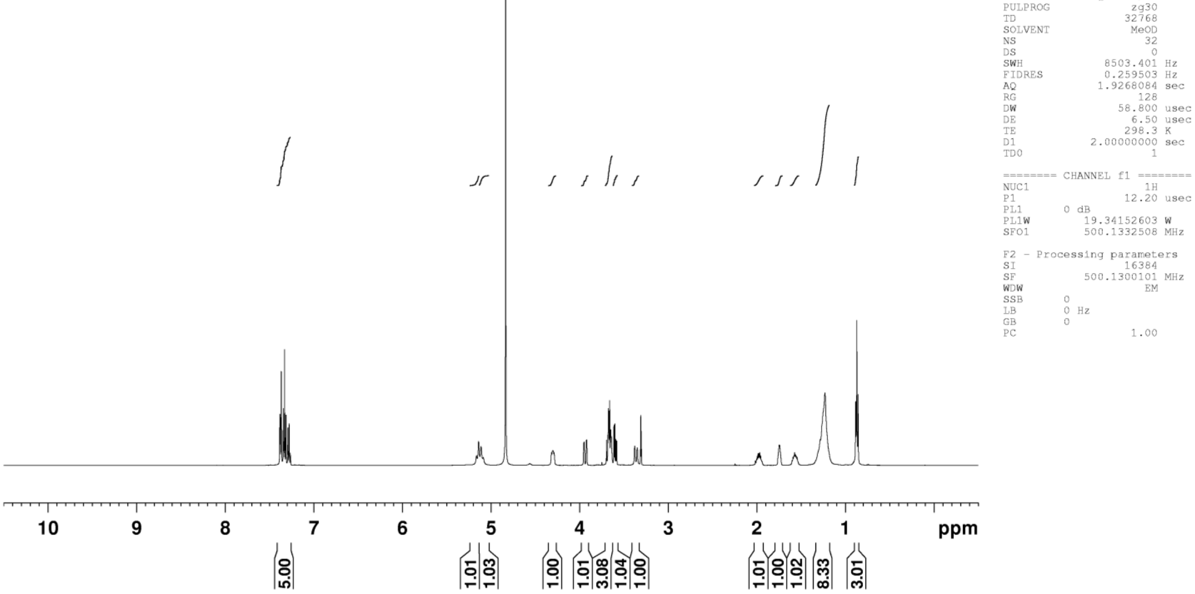

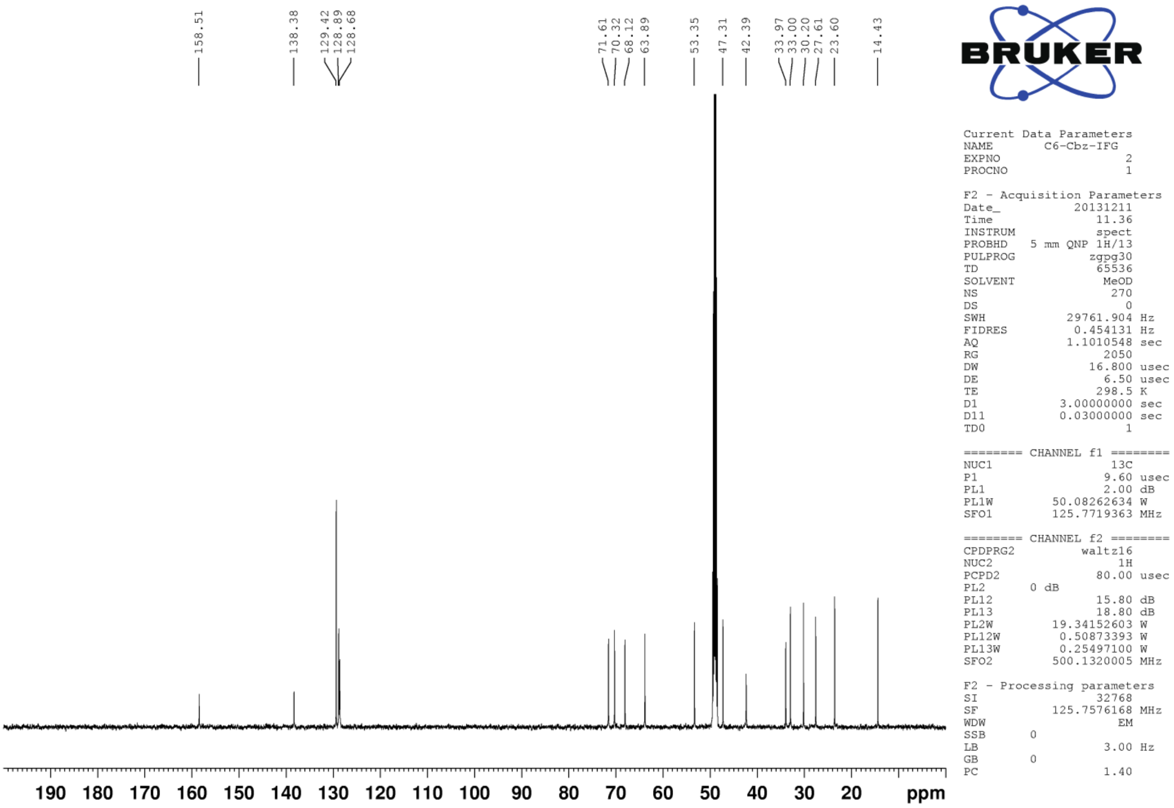

^{1}H-NMR spectrum ($CDCl_3$, 500 MHz) and ^{13}C-NMR spectrum ($CDCl_3$, 125 MHz) of **21**.

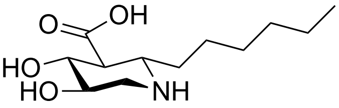

3

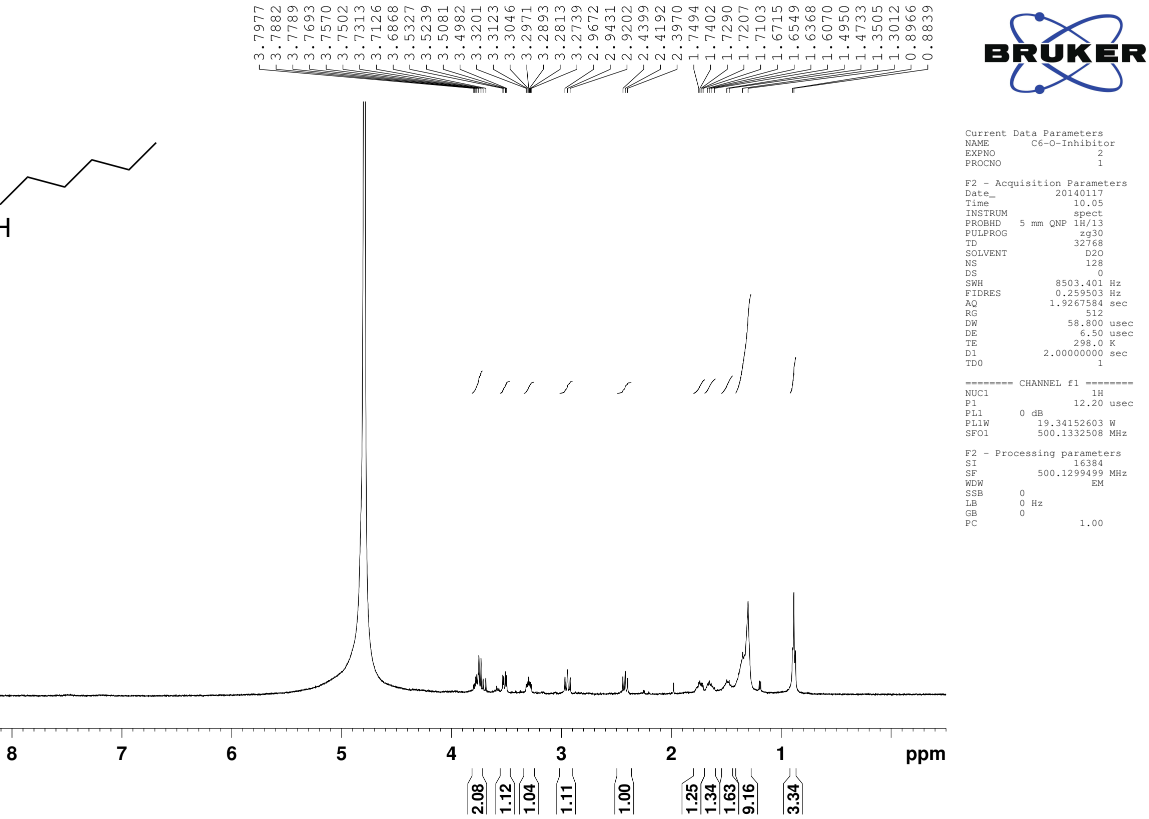

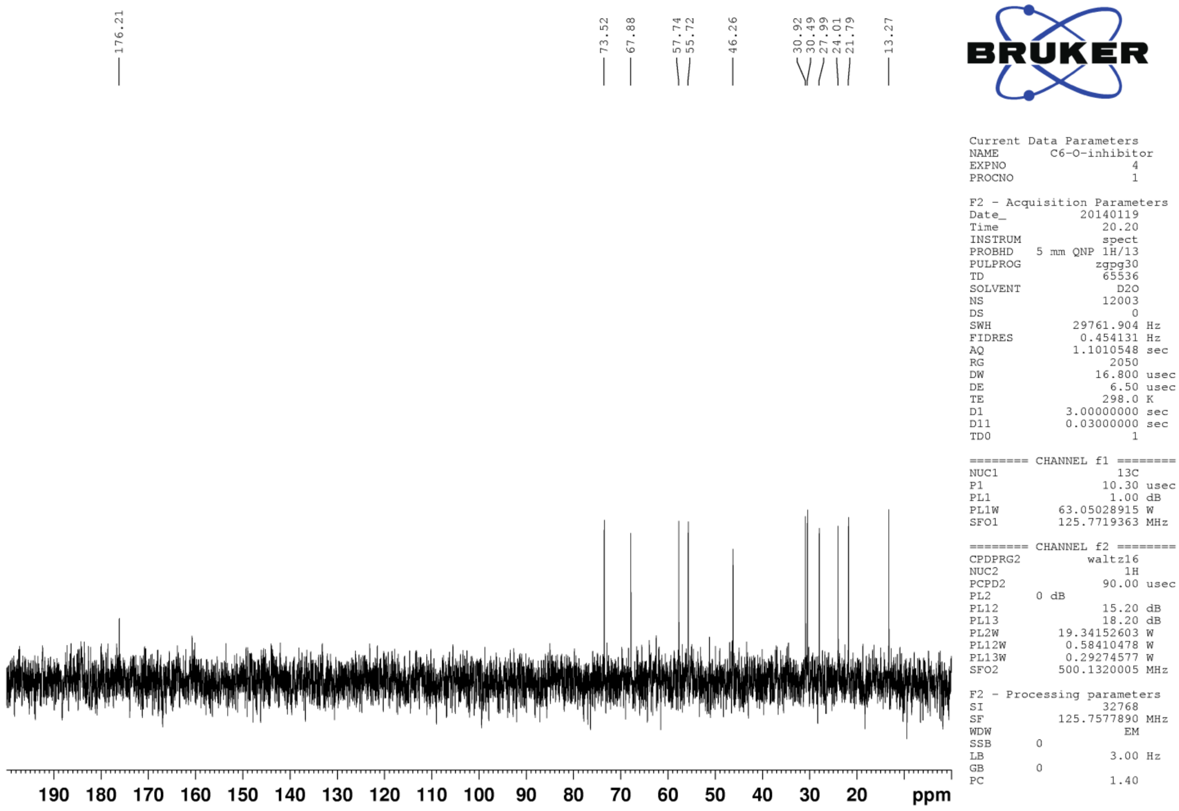

^{1}H-NMR spectrum ($CDCl_3$, 500 MHz) and ^{13}C-NMR spectrum ($CDCl_3$, 125 MHz) of **3**.

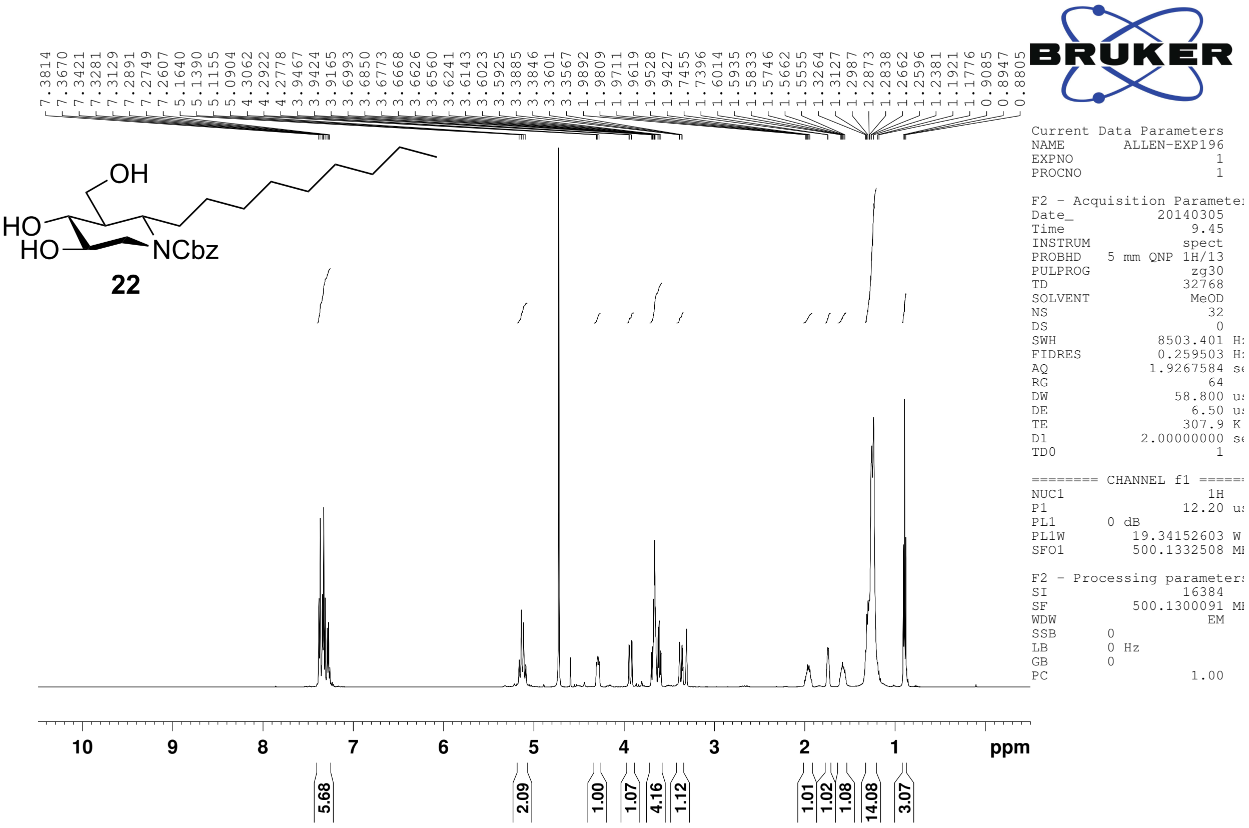

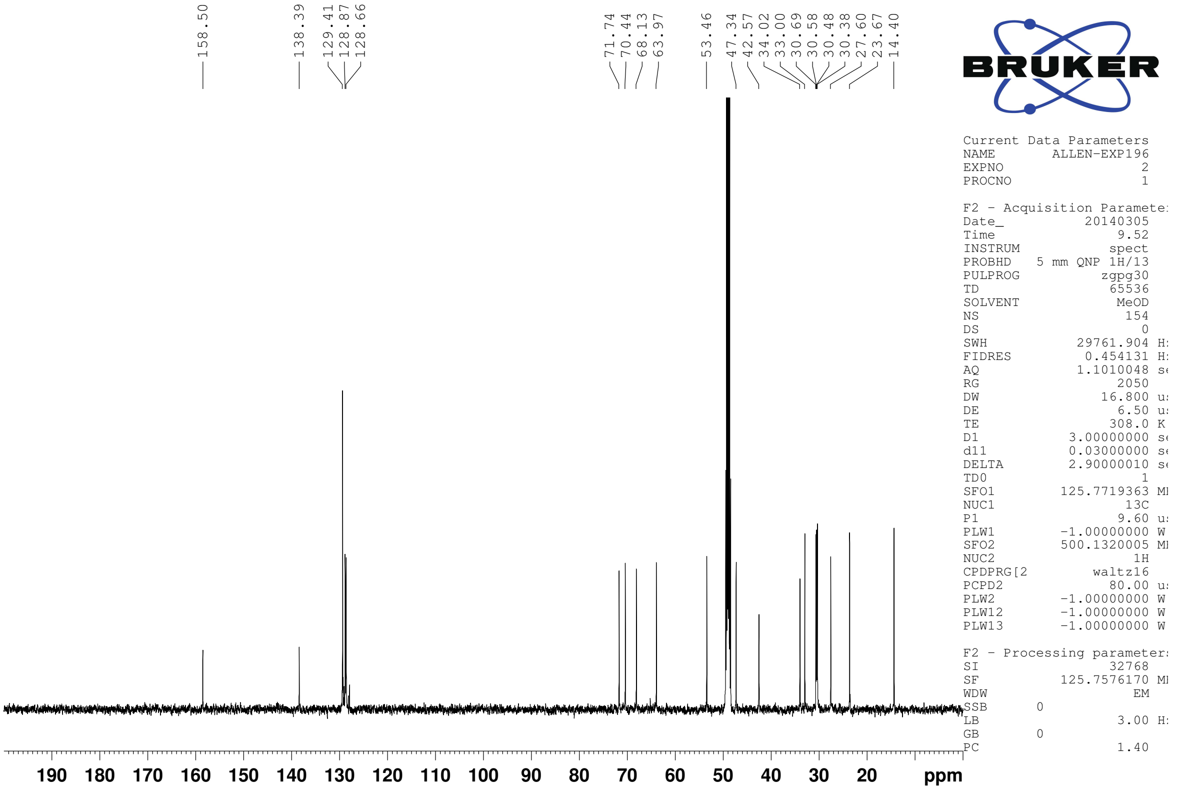

^{1}H-NMR spectrum ($CDCl_3$, 500 MHz) and ^{13}C-NMR spectrum ($CDCl_3$, 125 MHz) of **22**.

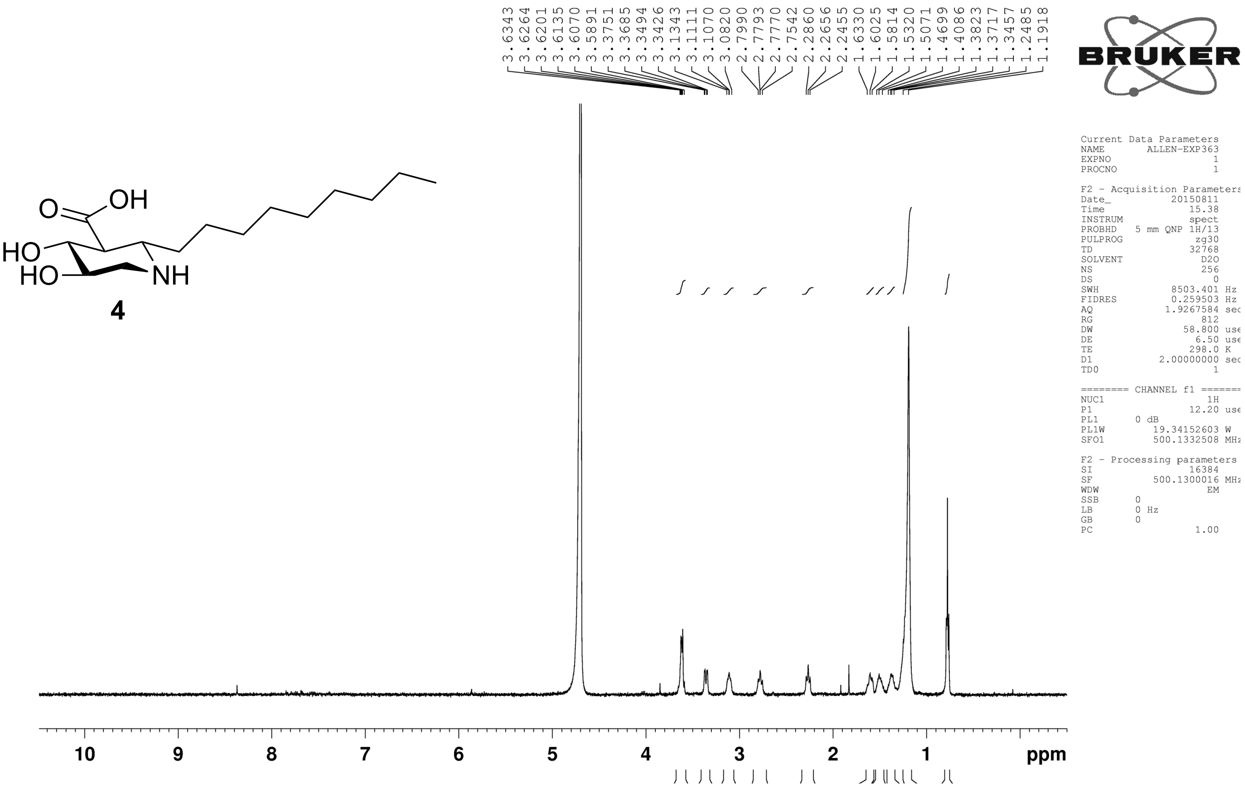

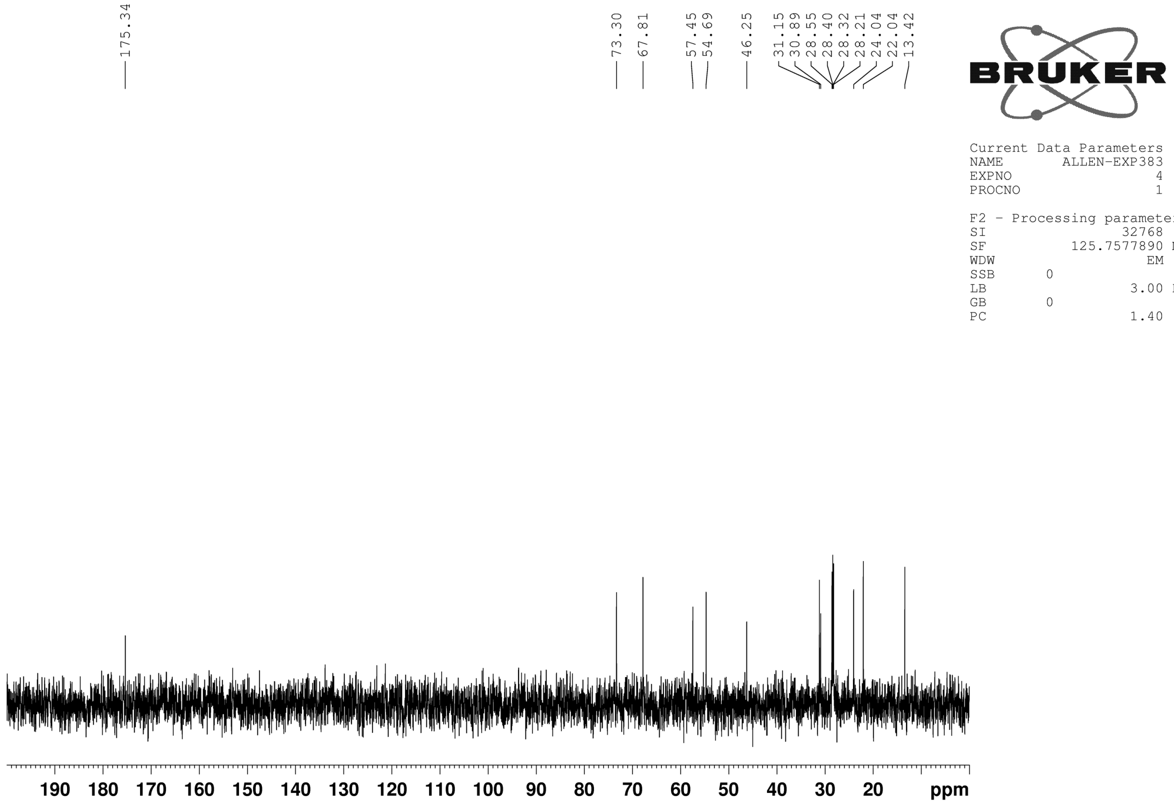

^{1}H-NMR spectrum ($CDCl_3$, 500 MHz) and ^{13}C-NMR spectrum ($CDCl_3$, 125 MHz) of **4**.

Supplementary information references

1. Waterhouse, A., Bertoni, M., Bienert, S., Studer, G., Tauriello, G., Gumienny, R., Heer, F.T., de Beer, T.A.P., Rempfer, C., Bordoli, L., Lepore, R. & Schwede, T. SWISS-MODEL: homology modelling of protein structures and complexes. *Nucleic Acids Res.* **46**, 296-303(2018).
2. E. D. Goddard-Borger & R. V. Stick, An expeditious synthesis of isofagomine. *Aust. J. Chem.* **60,** 211–213 (2007).
3. X. Zhu, K. A. Sheth, S. Li, H. H. Chang & J. Q. Fan, Rational design and synthesis of highly potent β-glucocerebrosidase inhibitors. *Angew. Chem. Int. Ed. Engl.* **44,** 7450–7453 (2005).
