# Supplementary material for: Entropy-driven binding of gut bacterial β-glucuronidase inhibitors ameliorates irinotecan-induced toxicity
Source: Commun Biol. 2021 Mar 4;4:280. doi: 10.1038/s42003-021-01815-w (PMC7933434; doi:10.1038/s42003-021-01815-w)
Supplement: Supplementary file 2 — Description of Additional Supplementary Files [file 42003_2021_1815_MOESM2_ESM.pdf]

Description of Additional Supplementary Files

File Name: Supplementary Data 1

Description: Data set for Table 1 (GUS activities was determined by the hydrolysis rate of 4-methylumbelliferyl-β-glucuronide)

File Name: Supplementary Data 2

Description: Data set for Figure 4 (including the definition of diarrhea scores, the record of diarrhea scores of mice from each group daily, and Welch's unpaired t-test of diarrhea score data)
