# Supplementary material for: Entropy-driven binding of gut bacterial β-glucuronidase inhibitors ameliorates irinotecan-induced toxicity
Source: Commun Biol. 2021 Mar 4;4:280. doi: 10.1038/s42003-021-01815-w (PMC7933434; doi:10.1038/s42003-021-01815-w)
Supplement: Supplementary file 5 — Reporting Summary [file 42003_2021_1815_MOESM5_ESM.pdf]

Corresponding author(s): Chun-Hung Lin

Last updated by author(s): Jan 22, 2021

Reporting Summary

Nature Research wishes to improve the reproducibility of the work that we publish. This form provides structure for consistency and transparency in reporting. For further information on Nature Research policies, see our Editorial Policies and the Editorial Policy Checklist.

Statistics

For all statistical analyses, confirm that the following items are present in the figure legend, table legend, main text, or Methods section.

n/a	Confirmed	
☐	☒	The exact sample size (*n*) for each experimental group/condition, given as a discrete number and unit of measurement
☐	☒	A statement on whether measurements were taken from distinct samples or whether the same sample was measured repeatedly
☐	☒	The statistical test(s) used AND whether they are one- or two-sided<br>*Only common tests should be described solely by name; describe more complex techniques in the Methods section.*
☐	☒	A description of all covariates tested
☐	☒	A description of any assumptions or corrections, such as tests of normality and adjustment for multiple comparisons
☐	☒	A full description of the statistical parameters including central tendency (e.g. means) or other basic estimates (e.g. regression coefficient) AND variation (e.g. standard deviation) or associated estimates of uncertainty (e.g. confidence intervals)
☐	☒	For null hypothesis testing, the test statistic (e.g. *F*, *t*, *r*) with confidence intervals, effect sizes, degrees of freedom and *P* value noted<br>*Give P values as exact values whenever suitable.*
☒	☐	For Bayesian analysis, information on the choice of priors and Markov chain Monte Carlo settings
☒	☐	For hierarchical and complex designs, identification of the appropriate level for tests and full reporting of outcomes
☒	☐	Estimates of effect sizes (e.g. Cohen's *d*, Pearson's *r*), indicating how they were calculated

Our web collection on statistics for biologists contains articles on many of the points above.

Software and code

Policy information about availability of computer code

Data collection	Software such as HKL2000,CCP4,PHENIX were used for structural data collection.
Data analysis	Data analysis Software such as WinCoot,PyMOL were used for refinement and structural data analysis. The software Origin and GraphpadPrism were used to analyze the experimental data.

For manuscripts utilizing custom algorithms or software that are central to the research but not yet described in published literature, software must be made available to editors and reviewers. We strongly encourage code deposition in a community repository (e.g. GitHub). See the Nature Research guidelines for submitting code & software for further information.

Data

Policy information about availability of data

All manuscripts must include a data availability statement. This statement should provide the following information, where applicable:

- Accession codes, unique identifiers, or web links for publicly available datasets
- A list of figures that have associated raw data
- A description of any restrictions on data availability

The data that support the findings of this study are available from the corresponding author on reasonable request. All the data supporting the findings of this study are available in the Supplementary Information. The source data underlying Table 1 and Figure 4b are provide as Supplementary Data 1 and 2. The coordinates of the crystal structures have been deposited to PDB (www.rcsb.org) and the entry numbers are 6LD6 (apo BdGUS), 6LDB (BdGUS/1), 6LDD (BdGUS/2), 6LDO (BdGUS/3), 6LDC (BdGUS/4), 6LEG (EcGUS/1), 6LEJ (EcGUS/2), 6LEL (EcGUS/3), and 6LEM (EcGUS/4).

Field-specific reporting

Please select the one below that is the best fit for your research. If you are not sure, read the appropriate sections before making your selection.

- [x] Life sciences
- [ ] Behavioural & social sciences
- [ ] Ecological, evolutionary & environmental sciences

For a reference copy of the document with all sections, see nature.com/documents/nr-reporting-summary-flat.pdf

Life sciences study design

All studies must disclose on these points even when the disclosure is negative.

Sample size	n/a,the sample size was chosen based on common practice in the field.
Data exclusions	No data were excluded from the analyses.
Replication	All attempts at replication were successful.
Randomization	n/a
Blinding	n/a

Reporting for specific materials, systems and methods

We require information from authors about some types of materials, experimental systems and methods used in many studies. Here, indicate whether each material, system or method listed is relevant to your study. If you are not sure if a list item applies to your research, read the appropriate section before selecting a response.

Materials & experimental systems

n/a	Involved in the study	
x		Antibodies
	x	Eukaryotic cell lines
x		Palaeontology and archaeology
	x	Animals and other organisms
x		Human research participants
x		Clinical data
x		Dual use research of concern

Methods

n/a	Involved in the study	
x		ChIP-seq
x		Flow cytometry
x		MRI-based neuroimaging

Eukaryotic cell lines

Policy information about cell lines

Cell line source(s)	All human cell lines were obtained from the American Type Culture Collection (ATCC)
Authentication	The cell lines were bought directly from commercial sources (ATCC). TCC uses morphology, karyotyping, and PCR based approaches to confirm the identity of cell lines.
Mycoplasma contamination	The cell lines were routinely tested for mycoplasma and were negative.
Commonly misidentified lines (See ICLAC register)	No misidentified cell lines.

Animals and other organisms

Policy information about studies involving animals; ARRIVE guidelines recommended for reporting animal research

Laboratory animals	Female BALB/c mice (8–12 weeks old) were purchased from the National Laboratory Animal Center (Taipei, Taiwan)
Wild animals	No wild animals were used
Field-collected samples	No field samples were acquired for this study
Ethics oversight	All experiments were done according to the standards of the United Kingdom Co-ordinating Committee on Cancer Research Guidelines for the Welfare of Animals in Experimental Neoplasia. All animal experiments were certified by Institute Animal Care and Use committee of Academia Sinica (ASIACUC), protocol ID: 12-07-384.

Note that full information on the approval of the study protocol must also be provided in the manuscript.
